# Cytokinin Inhibits Fungal Development and Virulence by Targeting the Cytoskeleton and Cellular Trafficking

**DOI:** 10.1128/mBio.03068-20

**Published:** 2021-10-19

**Authors:** Rupali Gupta, Gautam Anand, Lorena Pizarro, Dana Laor Bar-Yosef, Neta Kovetz, Noa Sela, Tal Yehuda, Ehud Gazit, Maya Bar

**Affiliations:** a Department of Plant Pathology and Weed Research, ARO, Volcani Institute, Rishon LeZion, Israel; b Institute of Agri-food, Animal and Environmental Sciences, Universidad de O’Higgins, Rancagua, Chile; c The Shmunis School of Biomedicine and Cancer Research, Tel Aviv University, Tel Aviv, Israel; INRA; Cornell University

**Keywords:** *Botrytis cinerea*, *Saccharomyces cerevisiae*, cytokinin, cell cycle, cytoskeleton, endocytosis

## Abstract

Cytokinin (CK) is an important plant developmental regulator, having activities in many aspects of plant life and response to the environment. CKs are involved in diverse processes in the plant, including stem cell maintenance, vascular differentiation, growth and branching of roots and shoots, leaf senescence, nutrient balance, and stress tolerance. In some cases, phytopathogens secrete CKs. It has been suggested that to achieve pathogenesis in the host, CK-secreting biotrophs manipulate CK signaling to regulate the host cell cycle and nutrient allocation. CK is known to induce host plant resistance to several classes of phytopathogens from a few works, with induced host immunity via salicylic acid signaling suggested to be the prevalent mechanism for this host resistance. Here, we show that CK directly inhibits the growth, development, and virulence of fungal phytopathogens. Focusing on Botrytis cinerea (*Bc*), we demonstrate that various aspects of fungal development can be reversibly inhibited by CK. We also found that CK affects both budding and fission yeast in a similar manner. Investigating the mechanism by which CK influences fungal development, we conducted RNA next-generation sequencing (RNA-NGS) on mock- and CK-treated *B. cinerea* samples, finding that CK alters the cell cycle, cytoskeleton, and endocytosis. Cell biology experiments demonstrated that CK affects cytoskeleton components and cellular trafficking in *Bc*, lowering endocytic rates and endomembrane compartment sizes, likely leading to reduced growth rates and arrested developmental programs. Mutant analyses in yeast confirmed that the endocytic pathway is altered by CK. Our work uncovers a remarkably conserved role for a plant growth hormone in fungal biology, suggesting that pathogen-host interactions resulted in fascinating molecular adaptations on fundamental processes in eukaryotic biology.

## INTRODUCTION

Cytokinins (CKs) are a class of extensively studied plant hormones, well known for their involvement in various aspects of plant life (1–4). CKs are involved in many physiological processes, such as stem cell control, vascular differentiation, senescence, chloroplast biogenesis, seed development, growth and branching of roots, shoots, and inflorescences, and senescence. CKs are also involved in nutrient balance and stress responses (5–8). Some findings have suggested a role for CKs in fungal pathogenesis (9–12). In some cases, plant pathogens can secrete CKs or induce CK production in the host plant. It has been suggested that to achieve pathogenesis in the host, CK-secreting biotrophs or hemibiotrophs manipulate CK signaling to regulate the host cell cycle and nutrient allocation (13). Exogenous application of CK was reported to reduce plant infection by powdery mildew (10), smut fungi (14), and viruses (15). Conidia, mycelia, and germinating uredospores of Puccinia graminis and P. recondita have been shown to accumulate CK, manipulating CK signaling to regulate the host plant cell cycle (16, 17).

High levels of CKs were found to increase plant resistance to bacterial and fungal pathogens (18–23). Different mechanisms have been suggested for this enhanced resistance. In *Arabidopsis*, it was suggested that CK-mediated resistance functions through salicylic acid (SA)-dependent mechanisms (20). An additional study suggested that CK signaling enhances the contribution of SA-mediated immunity in disease networks (24). More recently, CK-mediated immunity against Botrytis cinerea (*Bc*) in *Arabidopsis* was reported to be differentially modulated by jasmonate (JA) and ethylene (ET) (25). In tobacco, an SA-independent, phytoalexin-dependent mechanism was suggested (21). We recently reported that CK induces systemic immunity in tomato (Solanum lycopersicum), promoting resistance to fungal and bacterial pathogens (22, 23), including *Botrytis cinerea*, via an SA- and ET-dependent mechanism.

Given that CK can induce plant immunity and restrict phytopathogen growth in certain cases, direct effects of CK against phytopathogens are an intriguing possibility. A direct effect of CK on bacterial pathogens was ruled out in previous works (22, 24). Interestingly, high concentrations of exogenous CKs have been shown to stimulate conidial germination, but also inhibit germ tube growth, in species of *Erysiphe* that are obligate biotrophic powdery mildew-causing barley pathogens (26). High levels of CK were also reported to inhibit mycelial growth and pathogenesis of fungi in canola (9).

In this work, we investigated the direct effects of CK on fungal plant pathogens, using three phytopathogenic fungi with different lifestyles and infection modes. *B. cinerea* (gray mold), Sclerotium rolfsii (*Sr*; collar rot), and Fusarium oxysporum f. sp. *lycopersici* (*Fol*; Fusarium wilt) are widespread fungal plant pathogens that infect hundreds of plant species and cause huge losses every year (28–30). More than 50% of these losses occur in field-grown cultivated crops if synthetic/chemical management is not applied (31). Therefore, large amounts of chemical pesticides are widely required for the management of fungal pathogens. However, the extensive use of synthetic/chemical pesticides results in serious environmental pollution (32) and expedites the appearance of pesticide-resistant pathogens. To overcome this issue, there is a need to use chemicals that are natural, safe, and nontoxic to manage plant fungal disease.

Here, we demonstrate that CK directly inhibits the growth, development, and virulence of fungal plant pathogens. We found similar effects in a variety of plant-pathogenic fungi and in yeast. Using transcriptomics, cell biology, and classical fungal biology techniques, we investigated the fundamental and preserved effect of CK in fungal biology. We found that CK inhibits the cell cycle and affects the cytoskeleton and cellular trafficking. Our work uncovers a novel, remarkably conserved role for CK in fungal biology. Direct inhibition of *Bc* development and virulence by CK raises interesting questions regarding the possible roles of CK in plant-pathogen interactions, which we have partially addressed in this work.

## RESULTS

### Cytokinin directly inhibits *B. cinerea*, *S. rolfsii*, and F. oxysporum f. sp. *lycopersici*.

**FIG 1:**
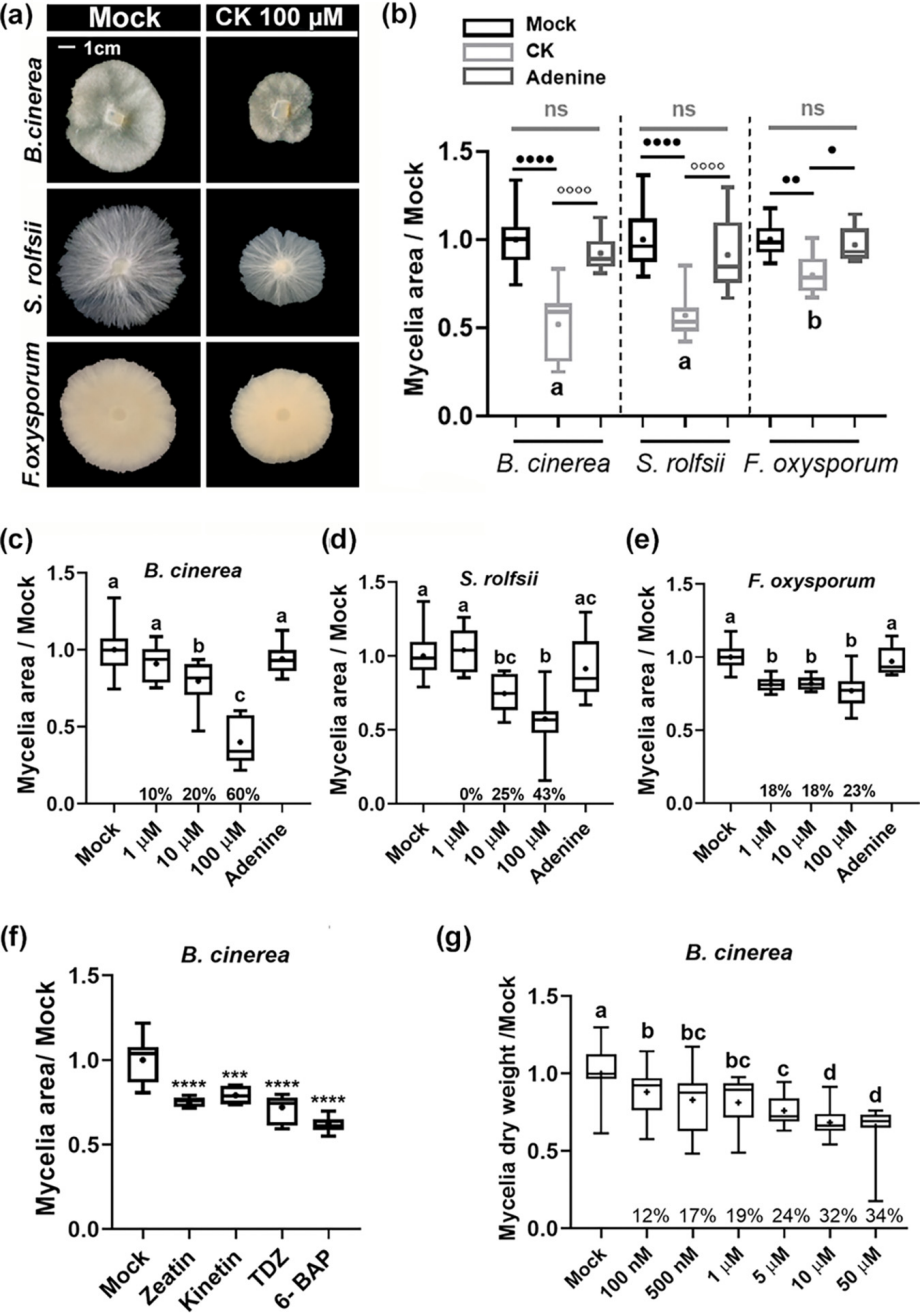
Direct effect of cytokinin on fungal growth. (a and b) *B. cinerea*, *S. rolfsii*, and F. oxysporum were cultured on potato dextrose agar (PDA) plates in the presence of 100 μM 6-benzylaminopurine (6-BAP) dissolved in 10 mM NaOH. (a) Representative pictures after 3 days. Bar = 1 cm. (b) Quantification of results from 4 to 6 biological repeats, including adenine (Ade, 100 μM) as a control (*n* > 20). Asterisks and dots (differences between mock [10 mM NaOH] and CK) and letters (differences between the level of CK growth inhibition in the different fungi) indicate significance in one-way ANOVA with a Bonferroni *post hoc* test. */^○^, *P *< 0.05; **/^○○^, *P  *< 0.01; ****/^○○○○^, *P* < 0.0001. ns, nonsignificant. (c to e) Dose response of *B. cinerea*, *S. rolfsii*, and F. oxysporum to CK (different concentrations of 6-BAP as indicated). Graphs represent 3 biological repeats ± SE (*n* > 6). Letters indicate significance in a one-way ANOVA. ****, *P < *0.0001 in all cases, with a Tukey *post hoc* test. (f) *B. cinerea* was cultured on PDA plates in the presence of a 100 μM concentration of the indicated CK compound (TDZ, thidiazuron). Quantification of results from 3 biological repeats (*n* = 12) is shown. Asterisks indicate significance in one-way ANOVA with a Tukey *post hoc* test. ***, *P < *0.001; ****, *P < *0.0001. (g) *B. cinerea* was cultured in liquid PDB in the presence of the indicated concentrations of 6-BAP. Quantification of results from 3 biological repeats (*n* = 6) is shown. Different letters indicate statistically significant differences in a *t* test with Welch’s correction (*P < *0.005). (b to g) Box plots display minimum to maximum values, with inner quartile ranges indicated by boxes and outer quartile ranges by whiskers. Lines indicate medians and dots indicate means.

In order to examine a possible direct effect of CK on fungal tomato pathogens, three fungi with varied lifestyles and infection modes were selected: *B. cinerea* (*Bc*), an airborne necrotrophic spore-producing ascomycete that causes gray mold disease in >1,400 hosts (33); *S. rolfsii* (*Sr*), a soilborne necrotrophic basidiomycete that does not produce spores and that causes southern blight disease in hundreds of hosts (34); and F. oxysporum f. sp. *lycopersici* (*Fol*), a soilborne hemibiotrophic ascomycete that causes Fusarium wilt disease in a host-specific manner (35). The effects of different CK concentrations and derivatives on the growth of *Bc*, *Sr*, and *Fol* mycelia are shown in Fig. 1. The cyclic synthetic CK 6-benzylaminopurine (6-BAP) (Fig. 1a, b, c, and g), the natural cyclic CKs zeatin and kinetin (Fig. 1f), and the synthetic bacterium-derived noncyclic CK thidiazuron (TDZ) (Fig. 1f) all inhibited the growth of *B. cinerea* on potato dextrose agar (PDA) plates. On plates, 6-BAP inhibited the growth of *Bc* at concentrations ranging from 10 μM (20% inhibition) to 100 μM (60% inhibition) (Fig. 1c). *Sr* growth inhibition at 10 μM (25%) and 100 μM (43%) was relatively similar to that of *Bc* (Fig. 1d). The least effect of 6-BAP was observed on *Fol*, ∼20% irrespective of CK concentration (Fig. 1e). In liquid media, concentrations ranging from 100 nM to 50 μM inhibited *Bc* mycelial growth in a dose-dependent manner, 12% to 34% (Fig. 1g).

To examine the breadth of this direct inhibition phenomenon, we tested the growth *in vitro* of additional phytopathogenic fungi in the presence of 100 μM 6-BAP or the control adenine. Our results show that CK directly inhibits mycelial growth of fungal pathogens from several different classes (ascomycetes and basidiomycetes) and different lifestyles (hemibiotrophs and necrotrophs) (see Fig. S1 in the supplemental material). All classes of phytopathogenic fungi tested were inhibited by CK (Fig. S1a); however, the level of inhibition also differed significantly among them, with Fusarium spp. showing the least inhibition (Fig. S1b). The phylogeny is detailed in Fig. S1c and does not indicate that the ability to be inhibited by CK is specific to any particular class or taxon.

10.1128/mBio.03068-20.2 FIG S1 Cytokinin inhibits fungal growth. Download FIG S1, PDF file, 0.9 MB. Copyright © 2021 Gupta et al. 2021 Gupta et al. https://creativecommons.org/licenses/by/4.0/ This content is distributed under the terms of the Creative Commons Attribution 4.0 International license.

We have previously reported that CK reduces tomato disease by inducing immunity (23). Fungal pathogens with different lifestyles have different infection and pathogenesis strategies, and host plants employ different protection mechanisms to resist different types of fungal pathogens. In order to examine the ability of CK to reduce disease caused by fungal pathogens which are directly inhibited by CK, we treated tomato plants with 100 μM CK (6-BAP) 24 h prior to pathogen inoculation. Figure S2 details the effect of CK in tomato disease caused by the different pathogens. 6-BAP pretreatment significantly decreased disease levels caused by the necrotrophic fungal pathogens *Bc*, as we previously reported (23), and *Sr*. Upwards of 50% disease reduction in tomato plants was observed with *Bc* and *Sr* compared to the control (Fig. S2a and b). However, no disease reduction was observed with *Fol* (Fig. S2c). To examine whether direct CK inhibition of *Bc* is reversible, we tested the virulence of spores grown in the presence of CK. We harvested *Bc* spores from fungi grown with or without CK, washed the spores, and normalized the spore count to 10^5^ spores/ml. We used equal amounts of mock-grown and CK-grown spores to infect tomato leaves. When spores harvested from mycelia grown with 6-BAP were used for infecting tomato leaves, no reduction in lesion size was observed when compared with spores grown without CK (Fig. S2d), indicating that although fewer spores developed in the presence of CK, they retained infective ability once the CK was removed.

10.1128/mBio.03068-20.3 FIG S2 Cytokinin inhibits on-plant pathogenesis of *B. cinerea* and *S. rolfsii* but not F. oxysporum. Download FIG S2, PDF file, 0.1 MB. Copyright © 2021 Gupta et al. 2021 Gupta et al. https://creativecommons.org/licenses/by/4.0/ This content is distributed under the terms of the Creative Commons Attribution 4.0 International license.

### Cytokinin inhibits *B. cinerea* sporulation, spore germination, and germ tube elongation.

**FIG 2:**
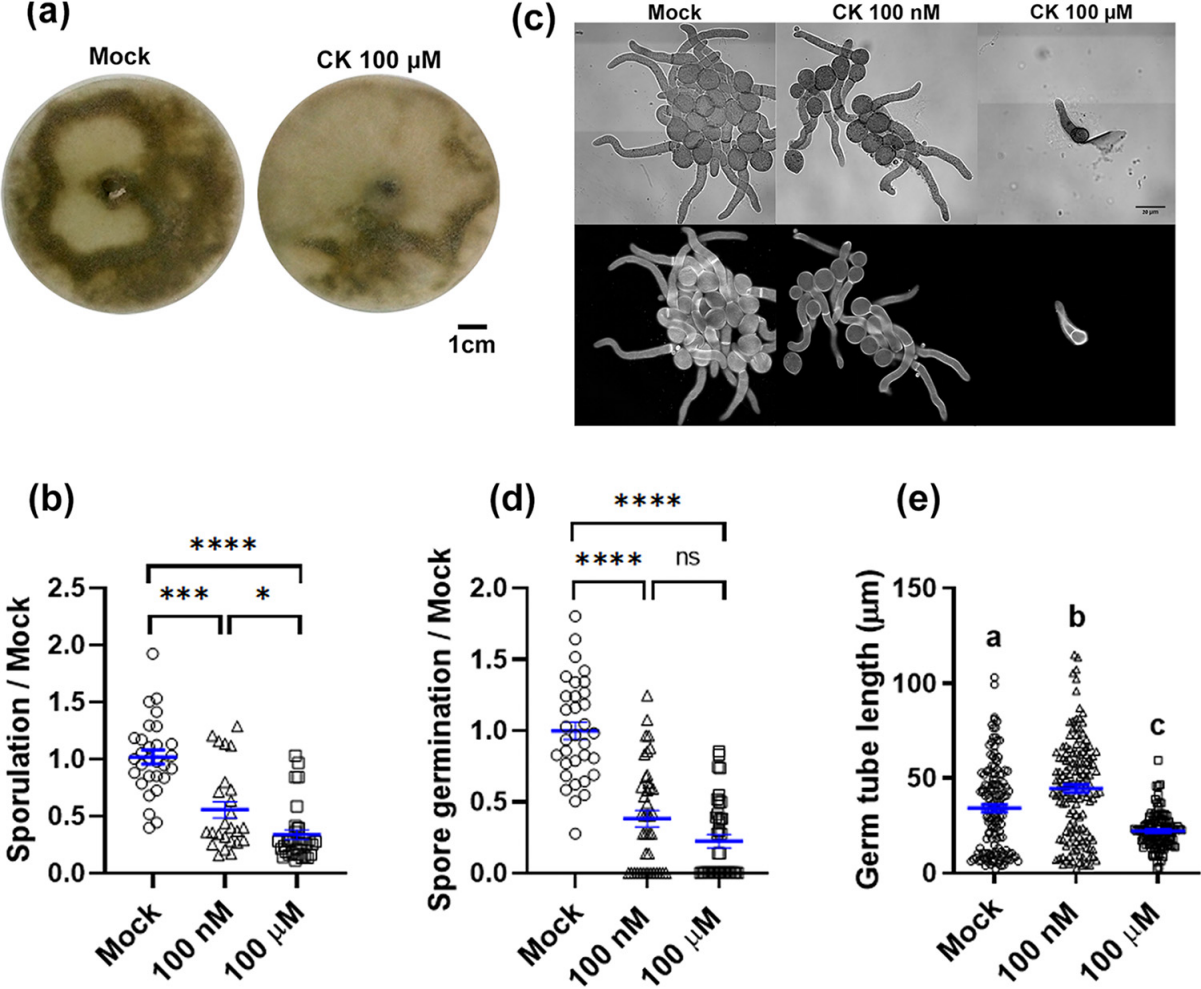
Cytokinin inhibits *Bc* sporulation and spore germination. (a and b) *B. cinerea* was cultured on PDA plates or liquid PDB in the presence of 100 nM or 100 μM 6-BAP. Spore formation is indicated by dark color (a) and quantified (b). (c to e) Spore germination and germ tube elongation are demonstrated (c) with calcofluor white staining (scale bar = 20 μm) and quantified (d and e). (b to e) Quantification of results from 4 biological repeats ± SE (*n* > 25). Asterisks or letters indicate significance in a Kruskal-Wallis ANOVA with Dunn’s *post hoc* test. *, *P < *0.05; ***, *P < *0.005; ****, *P < *0.001. Individual values are graphed, and blue bars represent SE.

Pursuant to the direct effect of CK on fungal mycelial growth, we studied its effect on additional aspects of fungal development, *viz*., sporulation, spore germination, and germ tube elongation. In samples treated with 100 nM CK, there were approximately 50% fewer spores produced than with mock treatment (Fig. 2a and b). The effect of CK on spore germination was even more pronounced (Fig. 2c and d). Fewer than 50% of the spores germinated with 100 nM CK. In addition to reduced spore germination in the presence of 6-BAP, the spores that did germinate had inhibited germ tube growth in 100 μM 6-BAP (Fig. 2e). After 8 h of growth with 100 μM 6-BAP, the germ tube length was 50% of the control (Fig. 2e), correlating with ∼50% inhibition of mycelial growth (Fig. 1a to c).

Liquid chromatography-mass spectrometry (LC-MS) hormonal measurements in mature tomato leaves demonstrate that they can contain 40 to 90 ng/g of active CKs (fresh weight) (Fig. S3a), as was previously reported for tomato leaves (36, 37). This amount roughly corresponds to 200 to 400 nM, which is above the amount (100 nM) which we found to have inhibitory effects on *B. cinerea* growth and development in liquid medium assays. This raises the possibility that fungal pathogens may be directly inhibited *in planta* by CK, particularly as several tested CK compounds were all found to inhibit *B. cinerea* mycelial growth (Fig. 1f), and we found *Bc* to activate the CK pathway (23). When investigating CK-mediated plant immunity, we had previously observed a reduction in active CKs 24 h after *Bc* inoculation (23), when no disease symptoms are yet visible on the plant. Recently, it was reported that *Bc* infection in *Arabidopsis* can increase the levels of some CKs, including *trans*-zeatin (*t*Z), and decrease the levels of other CKs, primarily ribosides (25). In *Arabidopsis*, the strongest increase in *t*Z was observed after 48 h (25). To further examine this, we assayed *t*Z levels 48 h after *Bc* inoculation, when active disease is present. The amount of *t*Z was increased by about 30% 48 h after *Bc* inoculation (Fig. S3b).

10.1128/mBio.03068-20.4 FIG S3 Quantification of active CKs in tomato leaves using LC-MS/MS. Download FIG S3, PDF file, 0.2 MB. Copyright © 2021 Gupta et al. 2021 Gupta et al. https://creativecommons.org/licenses/by/4.0/ This content is distributed under the terms of the Creative Commons Attribution 4.0 International license.

### Cytokinin is not toxic to *B. cinerea*.

**FIG 3:**
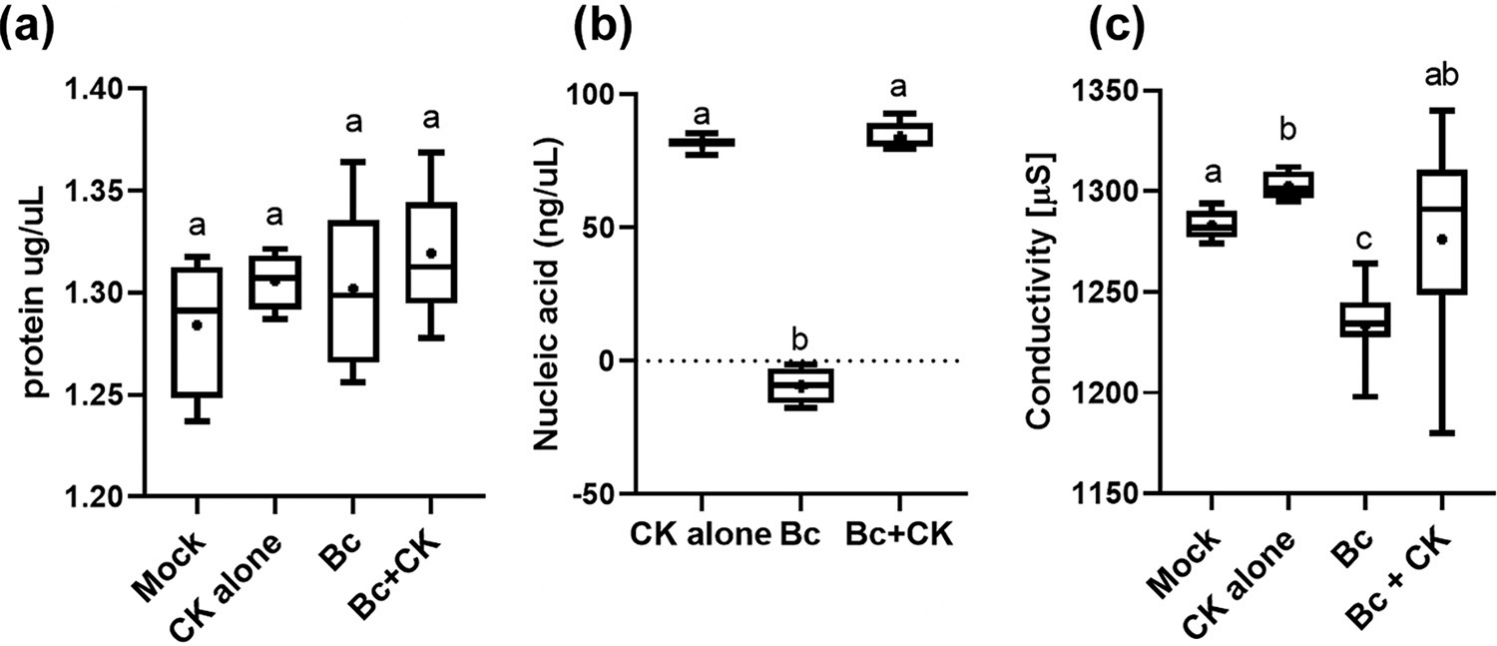
Cytokinin is not toxic to *B. cinerea*. *Bc* was cultured in liquid PDB, with or without 100 μM CK (6-benzylaminopurine). Mock, PDB; CK alone, CK in PDB without fungal matter. After 24 h, protein leakage (a), nucleic acid leakage (b), and medium conductivity (c) were measured. Graphs represent 3 biological repeats ± SE (*n*  > 6). Letters indicate significance in one-way ANOVA with Tukey’s *post hoc* test (a and c) or a Kruskal-Wallis ANOVA with Dunn’s *post hoc* test (b); a, ns; b and c, *P < *0.0001. Box plots with 2.5% whiskers are shown; lines indicate medians and dots indicate means. 6-BAP is a cyclic adenine-based molecule and has absorbance at 260 nm. No significant difference between control media containing CK alone (without *Bc*) or *Bc* with CK was observed in any of the parameters.

Disease assay results (Fig. S2) suggested that CK does not irreversibly harm fungal development. Spores generated from *Bc* grown in the presence of CK were able to infect tomato leaves normally once removed from the CK-containing environment. This indicated that the spores themselves were normal, and that disease reduction when infecting with spores in the presence of CK likely stems from a reduction in spore germination and germ tube growth, probably resulting in reduced penetration and invasive growth in the plant. To confirm that CK was not killing fungal cells in our experiments, we examined fungal cellular leakage in the presence of 100 μM CK. CK caused increases in nucleic acid and conductivity readings on its own in solution (potato dextrose broth [PDB]). Application of 100 μM 6-BAP did not lead to significant leakage of cellular contents of *Bc* (Fig. 3), as determined when the readings were compared with those of CK alone, without fungal material. No significant leakage of proteins were observed after 24 h of treatment with 100 μM 6-BAP, when compared with the control (Fig. 3a). Additionally, no nucleic acid leakage or change in electrical conductivity was observed upon 24 h of 100 μM 6-BAP treatment (Fig. 3b and c), when compared with CK alone, without fungal material. These results indicate that a 100 μM concentration of the CK 6-BAP is not toxic to *Bc*.

### Transcriptome profiling reveals pathways affected by CK in *B. cinerea*.

**FIG 4:**
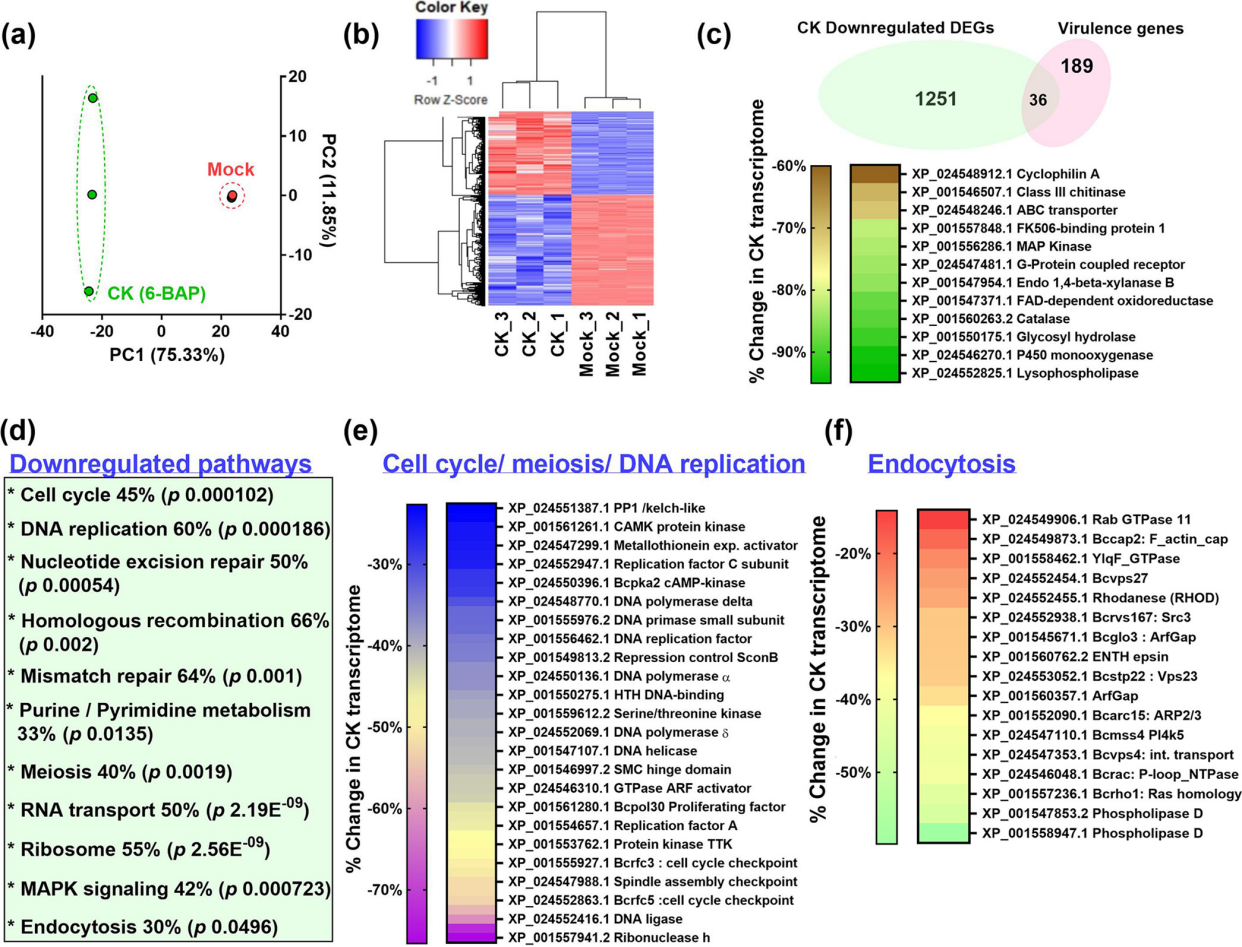
Transcriptomics reveal fungal pathways affected by CK. Shown is analysis of Illumina HiSeq NGS of *Bc* mock-treated or CK-treated samples, 3 biological repeats each. Gene expression values were computed as FPKM. Differential expression analysis was completed using the DESeq2 R package. Genes with an adjusted *P* value of no more than 0.05 and log_2_ fold change (FC) greater than 1 or lesser than −1 were considered differentially expressed. (a) Principal-component analysis (PCA) of 3 biological repeats from each treatment. PCA was calculated using the R function prcomp. (b) Heat map depicting the clustering of the different samples in terms of differentially expressed genes. Blue, negatively regulated; red, positively regulated. Color saturation indicates strength of differential expression. Heat map visualization was performed using R Bioconductor. See also Data Set S1A. (c) Analysis of virulence genes downregulated by CK treatment (*P < *0.036). Representative genes from different virulence groups are depicted in a heat map. See also Data Set S1D. (d) List of key statistically enriched pathways downregulated by CK. The KOBAS 3.0 tool was used to detect the statistical enrichment of differential expression genes in Kyoto Encyclopedia of Genes and Genomes (KEGG) pathways and Gene Ontology (GO). Pathways were tested for significant enrichment using Fisher’s exact test, with Benjamini and Hochberg FDR correction. Corrected *P* value was deemed significant at <0.05. The percentage of genes downregulated in each pathway and the corrected *P* value are indicated. See also Data Sets S1C and D. (e) Heat map representation of downregulated of genes belonging to the *Botrytis* cell cycle/meiosis/DNA replication pathways. (f) Heat map representation of downregulated genes belonging to the *Botrytis* endocytosis pathway.

To gain insight into the effects CK has on *Bc*, we conducted transcriptome profiling on *Bc* samples prepared from fungi grown with and without CK. RNA and library preparation, sequencing, and bioinformatics analysis methodologies are detailed in Materials and Methods. Principal-component analysis (PCA) demonstrated that the biological replicates were clustered well together (Fig. 4a), with mock samples being very similar and CK samples clustering together across PC1 (75%). The comparison yielded two clusters, exemplified in a heat map (Fig. 4b): genes downregulated by CK compared with mock treatment (bottom cluster) and genes upregulated by CK compared with mock treatment (top cluster). Individual genes having a log_2_ fold change of |1| or greater are provided in Data Set S1A. Distribution of Differentially Expressed Genes (DEGs) into various biological pathways was assessed using the Kyoto Encyclopedia of Genes and Genomes (KEGG). Downregulated KEGG pathways included various pathways related to the cell cycle and DNA replication (Fig. 4d), as well as endocytosis, mitogen-activated protein kinase (MAPK) signaling, and a variety of metabolic pathways (Fig. 4d). The full KEGG list with adjusted *P* values for each pathway is provided in Data Set S1B. Upregulated KEGG pathways included various pathways related to protein biosynthesis and processing, as well as the peroxisome and phagosome. The full KEGG list with adjusted *P* values for each pathway is provided in Data Set S1C. CK inhibited *Bc* infection (23) (Fig. S2). A “virulence” function is not annotated in KEGG or Gene Ontology (GO); therefore, to analyze the effect of CK on virulence, we generated our own *B. cinerea* virulence gene list. The list was generated using the database of virulence factors in fungal pathogens (38) and the published virulence gene groups according to Choquer et al. (39), and it contains 225 genes from 18 different functional groups. The downregulation of many genes involved in different aspects of *B. cinerea* life was reported to affect virulence (40); however, for our virulence gene list, we avoided genes that relate to fungal development and focused on genes reported to relate to the infection process. The full list is provided in Data Set S1D. We proceeded to analyze the representation of this gene group in the transcriptomic data, finding that virulence genes are significantly overrepresented in the group significantly downregulated by CK (*P < *0.036) (Fig. 4c). There was no change in the representation of virulence genes in the group significantly upregulated by CK (*P < *0.45). The representation factor (RF) was calculated as described previously (41). See Data Set S1D for further details.

10.1128/mBio.03068-20.1 DATA SET S1 (A) Genes having a significant log_(2)_Fold-change of |1| or greater in the *B. cinerea* CK transcriptome. (B) List of KEGG pathways significantly down-regulated by CK. (C) List of KEGG pathways significantly upregulated by CK. (D) List of virulence genes and their representation in the *B. cinerea* CK transcriptome. (E) List of cytoskeleton-related genes and their representation in the *B. cinerea* CK transcriptome. Download Data Set S1, XLSX file, 0.3 MB. Copyright © 2021 Gupta et al. 2021 Gupta et al. https://creativecommons.org/licenses/by/4.0/ This content is distributed under the terms of the Creative Commons Attribution 4.0 International license.

### Cytokinin affects the fungal cell cycle, cell morphology, and DNA replication.

**FIG 5:**
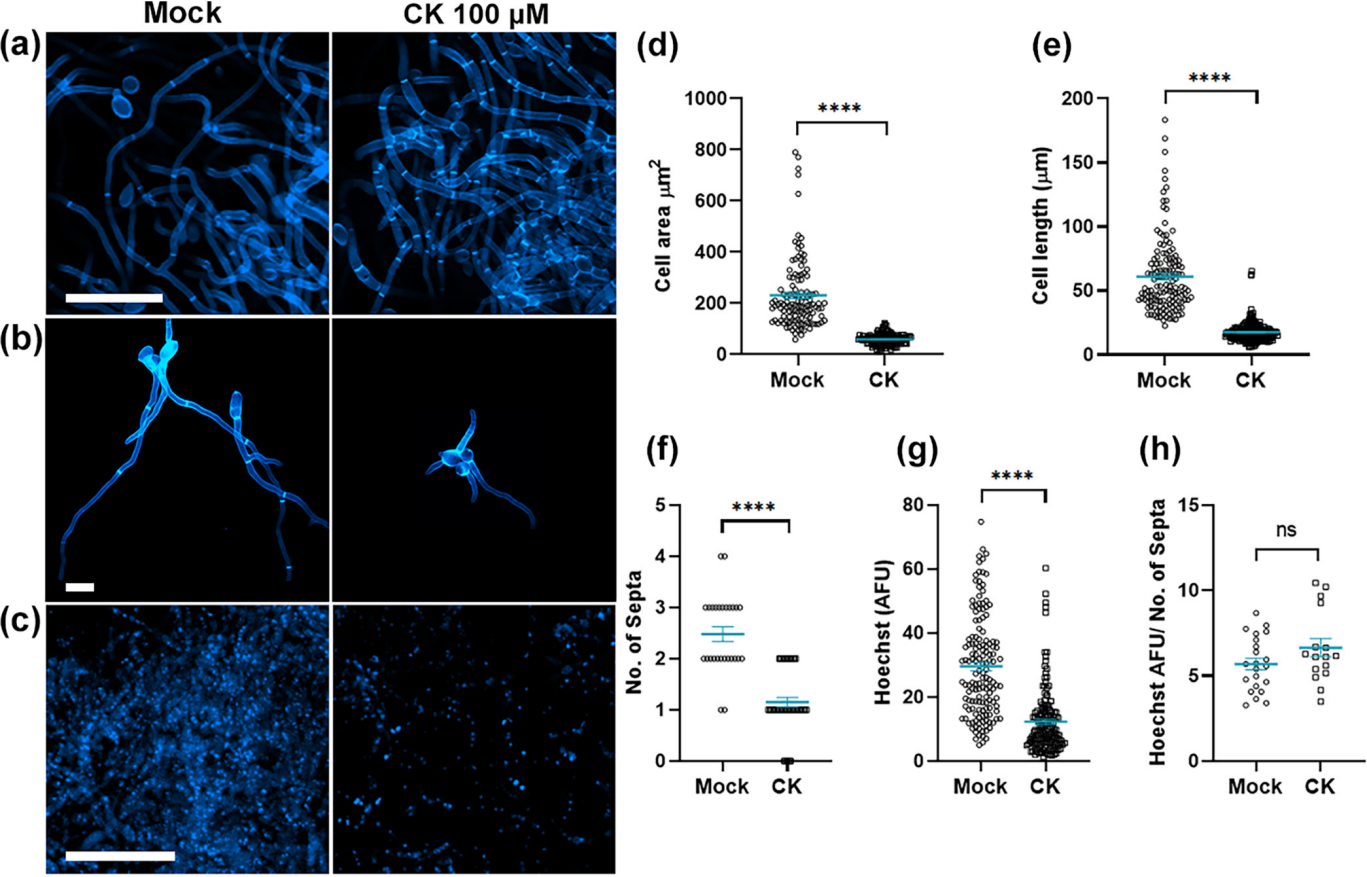
Cytokinin reduces *B. cinerea* cell elongation and DNA replication. *B. cinerea* was cultured in liquid PDB, with or without 100 μM CK (6-benzylaminopurine). After 8 h (f) or 16 h (a to e and h), growing hyphae were stained with calcofluor white (a, b, d, e, and f) or Hoechst (c, g, and h) and imaged on an Olympus IX 81 confocal laser scanning microscope using a 405-nm diode laser (1% power), under identical imaging conditions. (a and c) Bar = 50 μm; (b) bar = 20 μm. Cell area (d), distance between septa (e), number of septa in individual germinated hyphae (f), DNA staining (g), and the ratio between the amount of DNA staining and the number of septa in individual hyphae (h) were measured using Fiji-ImageJ. Graphs represent 3 to 6 biological repeats (*n* > 120 [d and e], *n* > 30 [f], *n* = 170 [g], and *n* = 16 [h]). Statistically significant differences between mock- and CK-treated samples were assessed using a Mann-Whitney U test. ****, *P < *0.0001. Individual values are graphed, and blue bars represent SE.

Since we observed that CK inhibits sporulation, spore germination, and hyphal growth, and the cell cycle and DNA replication were downregulated by CK treatment in the transcriptome analysis, we examined the morphology and relative DNA quantity of *Bc* cells grown with and without CK (Fig. 5). Using calcofluor white staining, we observed a significant reduction in the cell size upon CK (100 μM 6-BAP) treatment. Cell size was reduced to 25% in comparison with mock treatment, with cells appearing much smaller (Fig. 5a, b, and d). Similarly, the distance between the last two septa from the hyphal tip was reduced to about 30% of normal length (Fig. 5e). Quantifying the number of septa present in individual hyphae from discrete germinated spores demonstrated that 8 h after germination, CK-grown hyphae produced less than half the number of septa produced by mock treatment-grown hyphae (Fig. 5f), further confirming that cell replication is inhibited. Concurrently, we quantified Hoechst staining in mock treatment- and CK-grown mycelia. Hoechst binds to DNA and has been used before to estimate DNA content in live cell nuclei (42). CK-grown cells were stained by Hoechst at less than 50% of the amount of stain observed in mock treatment-grown cells (Fig. 5c and g). We further examined the amount of DNA per cell by calculating the amount of DNA (Hoechst) stain per number of septa in individual hyphae (Fig. 5h). The ratios of DNA per number of septa were similar in mock- and CK-treated cells, suggesting that the reduction in cell size may be a result of reduced DNA production. Images were taken under identical conditions. Reduction in Hoechst staining coupled with lower rates of cell replication, and the transcriptomic data indicating downregulation of cell cycle and meiosis pathways, indicates that CK may have inhibited cell division in *Bc*.

### Cytokinin interferes with the fungal cytoskeleton.

Our results demonstrate that CK inhibits fungal growth and development (Fig. 1, 2, 4, and 5). We hypothesized that CK affects a fundamental cellular process relevant to most fungi: a process that is crucial to execute the fast growth occurring in hyphal tips (43), growth that requires membrane remodeling (44). Based on the next-generation sequencing (NGS) results, we hypothesized that these affected processes, in addition to the cell cycle, are likely to be cytoskeletal integrity and/or cellular trafficking.

**FIG 6:**
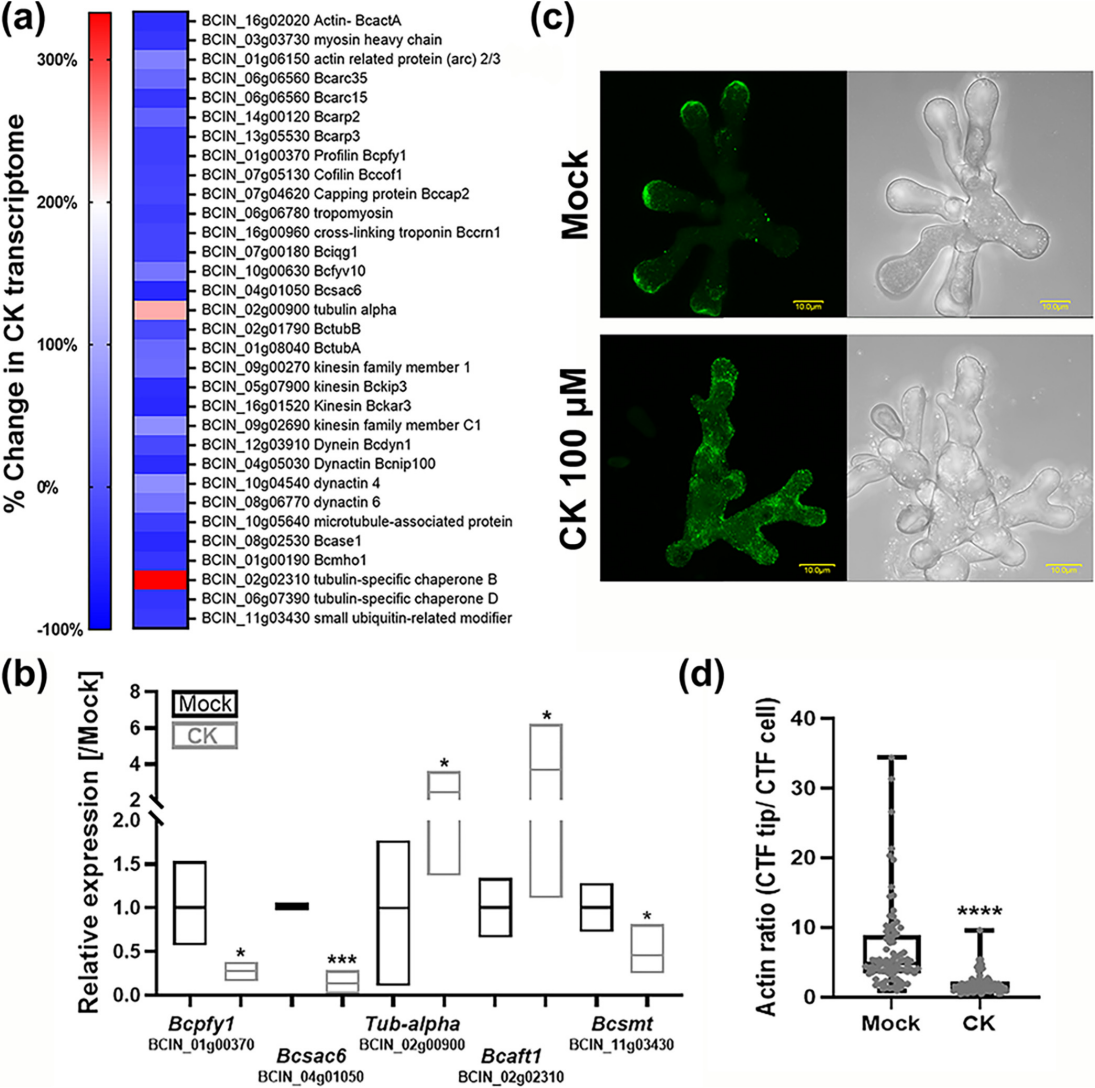
Cytokinin causes disorganization of the fungal cytoskeleton. (a) Cytoskeleton-related *B. cinerea* genes found to be differentially regulated in RNA-seq. See Data Set S1E for full list. (b) qRT-PCR validation of expression levels of 5 cytoskeleton-related genes: *Bcpfy1* (*profilin*; BCIN_01g00370), *Bcsac6* (*plastin-3*; BCIN_04g01050), *Bcsmt* (*small ubiquitin-related modifier*; BCIN_11g03430), *Tub-alpha* (*tubulin-alpha*; BCIN_02g00900), and *Bcaft1* (*tubulin-specific chaperone B*; BCIN_02g02310) upon CK treatment. *B. cinerea* was grown in PDB with the addition of 100 μM CK (6-BAP) or without (mock). Mock was set to 1. Gene expression values were normalized to a geometric mean of the expression of 3 housekeeping genes: the genes for ubiquitin-conjugating enzyme E2, iron transport multicopper oxidase, and adenosine deaminase. Floating bars represent minimum to maximum values of 3 biological repeats; line represents mean. Asterisks indicate significance in a two-tailed *t* test with Welch’s correction. *, *P < *0.05; ***, *P < *0.001. (c and d) *B. cinerea* was transformed with Lifeact-GFP. Germinated spores were treated with CK or not (mock) and grown for 6 h prior to confocal visualization. (c) Representative images. Bar = 10 μm. (d) Analysis of corrected total fluorescence (CTF) of the ratio between Lifeactin at the tip of the cell and the total cell in mock- and CK-treated cells. Three independent experiments were conducted with a total of 30 images analyzed (*n* > 80 growing hypha tips). Asterisks indicate significance in a Mann-Whitney U test. ****, *P < *0.0001.

To examine cytoskeleton integrity, we first validated the expression levels of cytoskeletal genes shown to be differential in the transcriptomic data. These genes are listed in Fig. 6a (saturated blue color indicates downregulation), with the full expression data provided in Data Set S1E. We independently confirmed relative expression of 5 genes from the data set by real-time quantitative reverse transcription PCR (qRT-PCR), selecting both downregulated (*Bcpfy1* [*profilin*; BCIN_01g00370], *Bcsac6* [*plastin-3*; BCIN_04g01050], and *Bcsmt* [*small ubiquitin-related modifier*; BCIN_11g03430]) and upregulated (*Tub-alpha* [*tubulin-alpha*; BCIN_02g00900] and *Bcaft1* [*tubulin-specific chaperone B*; BCIN_02g02310]) genes from the transcriptome (Fig. 6b). Consequently, we used the geometric mean of 3 housekeeping genes that are unrelated to the cytoskeleton for gene expression normalization. We transformed *B. cinerea* with Lifeact-green fluorescent protein (GFP) (45) and proceeded to treat the transformed fungal cells with CK. We observed mislocalization of actin, which is normally localized to growing hyphal tips (46, 47), upon CK treatment. CK caused F-actin to be distributed more uniformly throughout the cells and to lose most of its tip-specific localization (Fig. 6c and d). Analysis of corrected total fluorescence in mock- and CK-treated cells demonstrated that the ratio between actin in the tip of the cell and the total cell decreased greatly in the presence of CK (Fig. 6d). The transformed fungus showed the characteristic hyperbranched-hyphae phenotype of Lifeact overexpression (45) in both mock- and CK-treated samples.

### Cytokinin inhibits fungal endocytosis.

**FIG 7:**
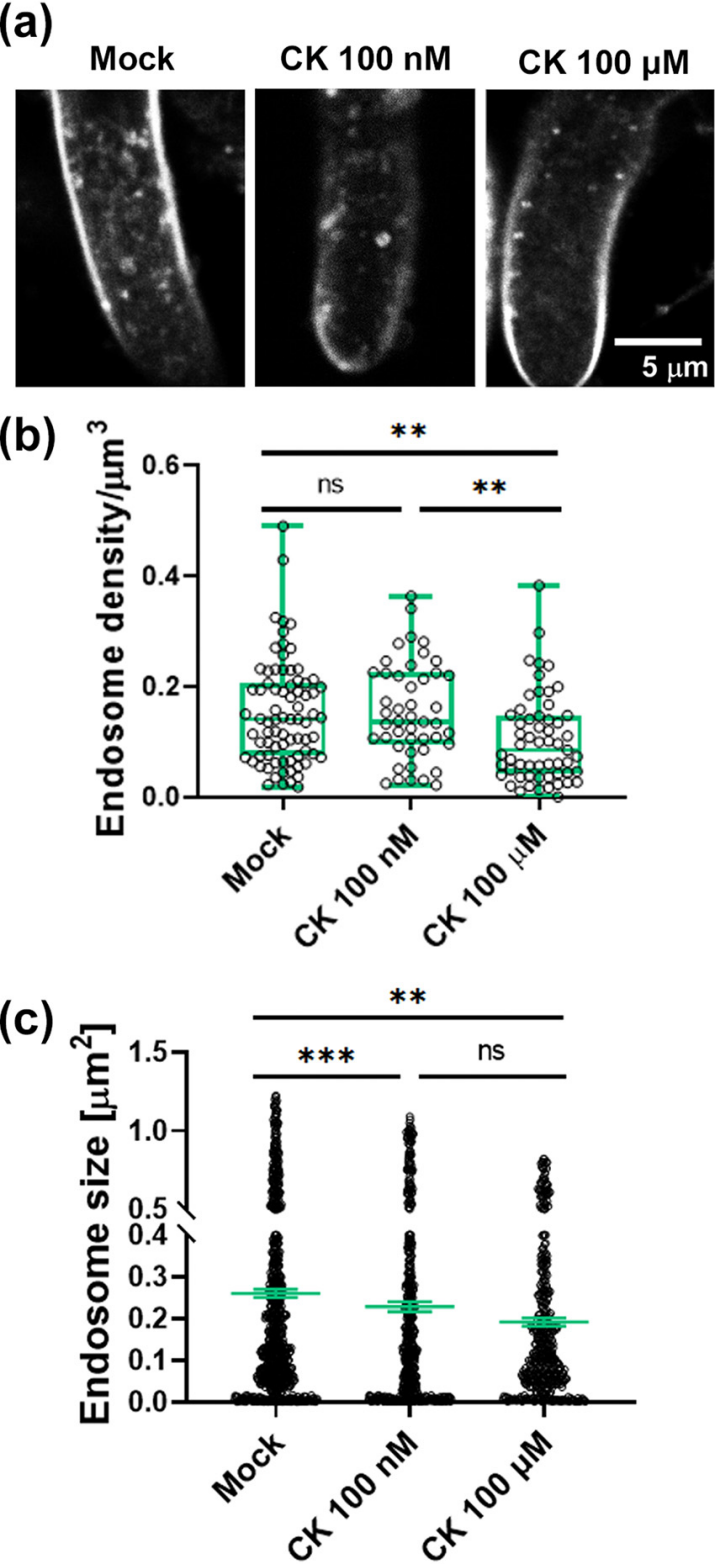
Cytokinin inhibits FM4-64 endocytosis in growing *B. cinerea* hyphae. *Bc* was cultured in liquid PDB in the presence of 100 nM or 100 μM CK (6-benzylaminopurine) for 16 h. (a) FM4-64 endocytic vesicles in *Bc* hyphae; (b) quantification of the amount of endocytic vesicles; (c) quantification of the average size of vesicles. Measurements were done using the counting tool of Fiji. Quantification of results from 7 biological repeats is shown. (b) *n* > 45; box plot with all values displayed. Boxes indicate inner quartile ranges, with lines indicating medians and whiskers indicate outer quartile ranges. (c) *n* > 450; all values are displayed, and line indicates SE. Asterisks indicate significance in Kruskal-Wallis ANOVA with Dunn’s *post hoc* test. **, *P < *0.01; ***, *P < *0.001.

We and others (23, 48) have previously shown that CK can influence cellular trafficking in plants. Further to our results demonstrating that CK causes mislocalization of the cytoskeleton in *Bc*, and since the endocytic pathway was also found to be significantly downregulated by CK in the transcriptomics (Fig. 4), we examined the effect of CK on endocytosis in *Bc*. Figure 7 shows that 6-BAP inhibits endocytosis of the endocytic tracer FM4-64, which is routinely used in fungi (49), reducing the amount of endocytic vesicles by more than 50% (Fig. 7a and b). 6-BAP also caused a significant decrease in the size of the vesicles containing endocytic tracer (Fig. 7c), at both the 100 nM and 100 μM concentrations, similar to its effect on sporulation and spore germination (Fig. 2). This suggests that in parallel with the effect on the cytoskeleton, CK has a possible impact on membrane function and/or fission of vesicles. Examination of the spitzenkorper (Spk) structure (50) in mock- and CK-treated cells also showed that the Spk region contained less FM-464 staining upon 100 nM and 100 μM 6-BAP treatment (Fig. S4), correlating with the cytoskeleton mislocalization (Fig. 6) and endocytosis inhibition (Fig. 7), and suggesting that impaired delivery of vesicles to the Spk and impaired Spk function also underlie reduced hyphal growth.

10.1128/mBio.03068-20.5 FIG S4 Cytokinin affects the integrity of the spitzenkorper. Download FIG S4, PDF file, 0.4 MB. Copyright © 2021 Gupta et al. 2021 Gupta et al. https://creativecommons.org/licenses/by/4.0/ This content is distributed under the terms of the Creative Commons Attribution 4.0 International license.

### Depolymerization of the cytoskeleton affects *B. cinerea* CK sensitivity.

We examined combined effects of CK and cytoskeleton disruption, using benomyl (Ben) and latrunculin B (LatB). Benomyl depolymerizes microtubules and has been previously used as a fungicide and in studies of fungal cell cycle and cytoskeleton (51–53). Latrunculin B depolymerizes actin filaments and has also previously been used for cytoskeletal studies in fungi (54). We assayed the combined effect of CK and Ben or LatB on endocytosis, assaying endosome size and density. We observed that CK and Ben (Fig. S5Aa and c) and CK and LatB (Fig. S5Ab and d) affect endocytic compartments in similar manners, finding no enhancement of endocytosis inhibition when 100 μM CK was combined with either drug at 1 μM (Fig. S5A), suggesting that CK may inhibit endocytosis in part through its effect on the cytoskeleton, though downregulation of endocytic genes is also present (Fig. 4).

10.1128/mBio.03068-20.6 FIG S5 (A) Inhibition of the cellular cytoskeleton affects *B. cinerea* cytokinin sensitivity-cellular trafficking. (B) Additive effects of CK and cytoskeleton inhibitors on fungal growth. Download FIG S5, PDF file, 0.4 MB. Copyright © 2021 Gupta et al. 2021 Gupta et al. https://creativecommons.org/licenses/by/4.0/ This content is distributed under the terms of the Creative Commons Attribution 4.0 International license.

We further examined the combined effect of CK and Ben or LatB on growth inhibition of fungal mycelia. Figure S5B demonstrates that combined effects of CK and cytoskeleton inhibitors are dependent on applied concentrations. When the cytoskeleton is strongly depolymerized (above 500 nM Ben or 10 nM LatB), Ben- and LatB-mediated growth inhibition is not further enhanced by the addition of CK (Fig. S5BA and B). However, when the cytoskeleton-inhibiting drugs were applied at lower concentrations, an additive effect of CK was observed (Fig. S5BA to D). This suggests that CK may have a partially overlapping effect with benomyl or LatB on the cytoskeleton/cell cycle.

### Cytokinin reduces yeast growth and endocytosis.

**FIG 8:**
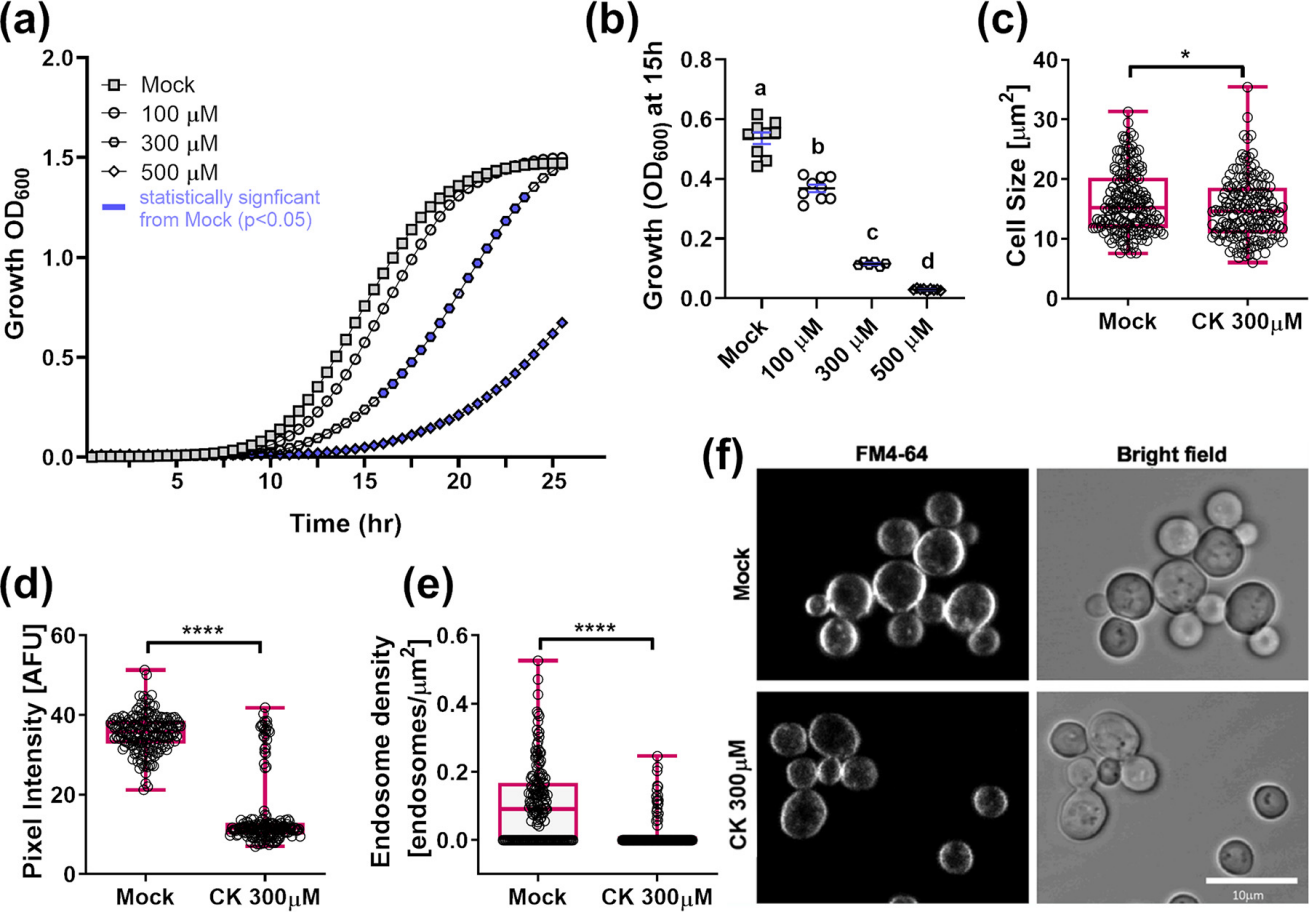
Cytokinin inhibits growth and endocytosis in budding yeast. (a) Wild-type Saccharomyces cerevisiae was grown overnight at 30°C in minimal synthetic defined medium, treated with either 10 μM NaOH (mock) or the addition of indicated concentrations of CK (6-benzylaminopurine). Cells were incubated at 30°C for 25 h, with continuous shaking. Average growth per time point for 3 experiments is presented (*n* = 9). Blue symbols indicate statistically significant difference from mock treatment in a two-tailed *t* test with Holm-Sidak correction (*P < *0.05). (b) Average growth (OD) at mid-log phase (15 h) in three independent experiments. Letters indicate significance in a one-way ANOVA with a *post hoc* Tukey test (*P < *0.0001). All points are displayed; red lines indicate SE. (c to f) S. cerevisiae yeast cells were grown overnight at 30°C in YPD medium, diluted (OD_600_ = 0.2), and incubated for 6 h in YPD media (mock) or media supplemented with 300 μM CK (6-benzylaminopurine). Cells were incubated with 24 μM FM4-64 (Invitrogen) at 4°C for 30 min. Subsequently, the FM4-64-containing medium was replaced with fresh medium and cultures was incubated at 28°C for 15 min. Confocal microscopy images were acquired using a Zeiss LSM780 confocal microscope. (c) Cell size; (d) total internalized FM4-64 per cell represented by pixel intensity; (e) endosome density (f) representative images. Bar, 10 μm. Box plots have all values displayed; line indicates median. (c to e) *n* > 160 cells. Image analysis was performed using Fiji-ImageJ with raw images collected from 3 independent biological experiments, on a defined region of interest that excluded the plasma membrane. Endosome counts were done with the 3D object counter tool, and pixel intensity was measured using the measurement analysis tool. Asterisks represent statistical significance in a Mann-Whitney U test. *, *P < *0.05; ****, *P < *0.0001.

Given our observations that CK can affect as fundamental a cellular process as endocytosis, we examined inhibitory roles for CK in the growth of Saccharomyces cerevisiae, a budding yeast, and Schizosaccharomyces pombe, a fission yeast, the growth of which more closely resembles that of fungal hyphae. Growth curves were generated by measuring optical density at 600 nm (OD_600_) over time, as previously described (55). We observed that CK inhibits the growth of S. cerevisiae (Fig. 8a and b) and S. pombe (Fig. S6Aa and b) in a dose-dependent manner. We found that S. pombe was more strongly inhibited. Interestingly, *trans*-zeatin was previously reported not to affect S. pombe cell division (56).

10.1128/mBio.03068-20.7 FIG S6 (A) Cytokinin inhibits the growth of fission yeast. (B) Cytokinin inhibits FM4-64 endocytosis in fission yeast. Download FIG S6, PDF file, 0.6 MB. Copyright © 2021 Gupta et al. 2021 Gupta et al. https://creativecommons.org/licenses/by/4.0/ This content is distributed under the terms of the Creative Commons Attribution 4.0 International license.

To examine whether growth inhibition is mediated by endocytosis in yeast, as we found for *Bc*, we conducted endocytic assays in S. cerevisiae (Fig. 8) and S. pombe (Fig. S6B). Yeast cultures were grown overnight, diluted to an OD_600_ of 0.2, and grown for a further 6 h with or without CK. Cultures were then stained with FM4-64 (57). We found that CK reduces cell size, internalization of FM4-64, endosome size, and endosome density in both S. cerevisiae (Fig. 8c to f) and S. pombe (Fig. S6Ba to e), indicating that endocytosis is likely a universal mechanism through which CK exerts its effect.

**FIG 9:**
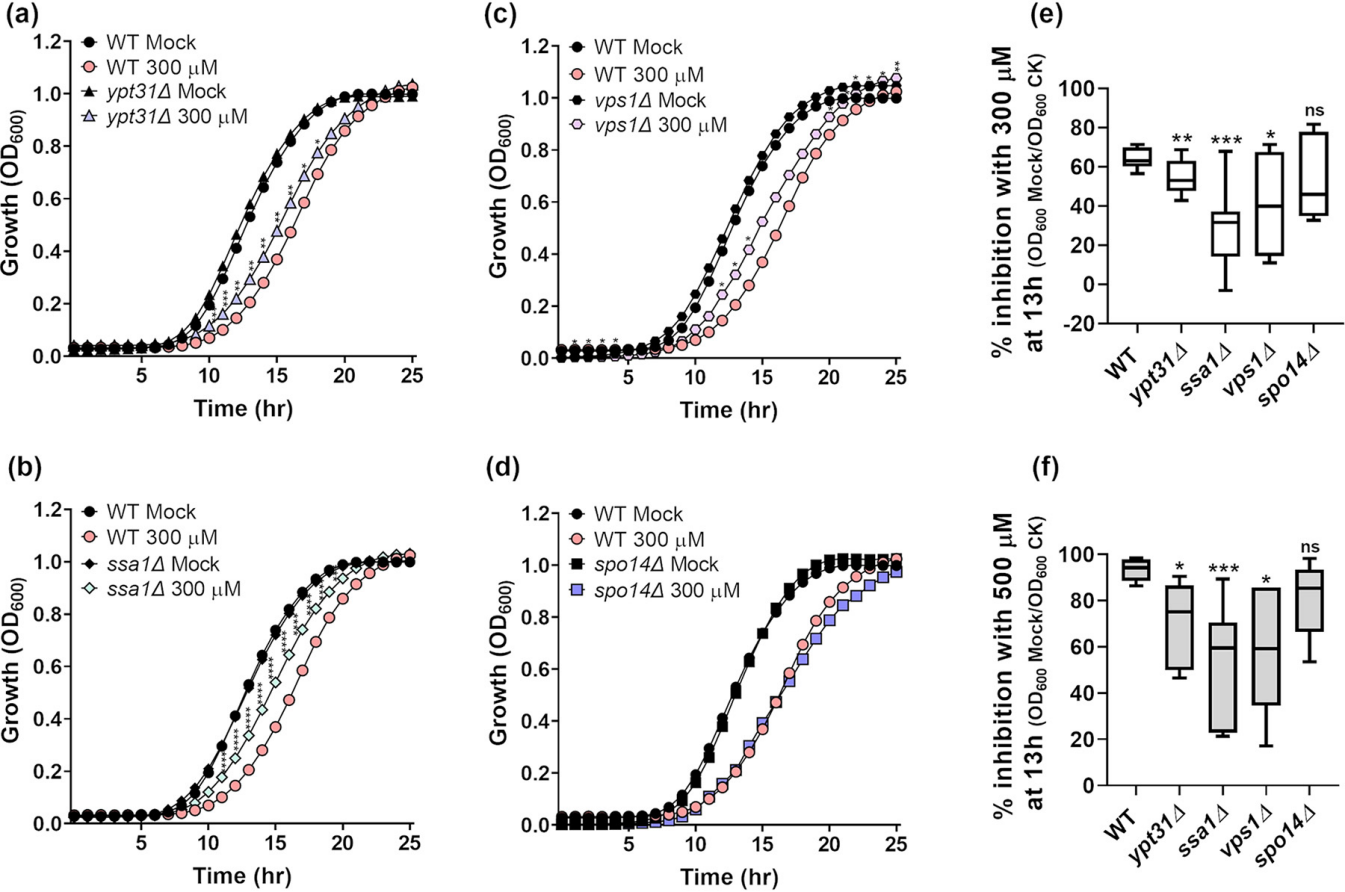
Cytokinin-mediated growth inhibition is partially rescued in budding yeast endocytic mutants. S. cerevisiae wild-type (WT; a to f) and *ypt31Δ* (a, e, and f), *ssa1Δ* (b, e, and f), *vps1Δ* (c, e, and f), and *spo14Δ* (d, e, and f) mutant strains were grown overnight at 30°C for 25 h, in minimal synthetic defined medium treated with either 10 μM NaOH (mock) or the addition of 300 μM (a to e) or 500 μM (f) CK (6-benzylaminopurine). (a to d) Average growth per time point for three experiments is presented (*n* = 9). Asterisks indicate statistical significance of each mutant with CK compared to WT with CK, in a two-tailed *t* test with Holm-Sidak correction. *, *P* < 0.05; **, *P* < 0.01; ***, *P* < 0.001; ****, *P* < 0.0001. (e and f) Percentage of growth inhibition of each strain with 300 μM CK (e) and 500 μM CK (f) compared to mock treatment, at 13 h, in three independent experiments (*n* = 9). Box plots are shown with interquartile ranges (box), medians (black line in box), and outer quartile whiskers (minimum to maximum values). Asterisks indicate significance in a one-way ANOVA with a Tukey *post hoc* test. *, *P < *0.05; **, *P < *0.01; ***, *P < *0.001.

In parallel, the effect of CK on the growth of S. cerevisiae endocytic mutants was examined. S. cerevisiae homologs of genes downregulated by CK in *Bc*, known to be involved in endocytosis, and the absence of which is known not to be lethal, were selected. S. cerevisiae knockout mutant strains were constructed by disrupting the indicated genes as described in Materials and Methods. Mutations in YPT31, a family 11 Rab/GTPase known to be involved in vesicular trafficking (58), SSA1, known to be involved in clathrin vesicle uncoating, intracellular transport, and protein folding (59), *VPS1*, a dynamin-like GTPase known to be required for vacuolar sorting, cytoskeleton organization and endocytosis (60, 61) and SPO14, a phospholipase D protein required for sporulation (62), were generated and examined. *Botrytis* homologs of the corresponding genes, which are downregulated by CK, can be found in Fig. 4f. Three out of the four generated mutants, the *ypt31Δ*, *ssa1Δ*, and *vps1Δ* mutants, exhibited a partial rescue in CK-mediated inhibition (Fig. 9a, c, e, and f), growing significantly better in the presence of 300 and 500 μM 6-BAP than the wild-type (WT) strain. The *spo14Δ* mutation did not rescue CK-mediated growth inhibition (Fig. 9d to f).

## DISCUSSION

As reported previously by us and others, and as shown here (Fig. S1), CK promotes fungal disease resistance in plants (20, 23). Direct effects of CK on fungal growth and development have not been investigated in depth in plant-free systems, and although a few studies have discussed some effects of CK on fungal pathogen growth (9, 10), no mechanisms for CK antifungal activity have yet been reported. The present study was performed to examine the direct inhibitory effect of CK on fungal phytopathogens. We focused our efforts on three pathogens, *Bc*, *Sr*, and *Fol*, with varied lifestyles and infection modes: *Bc*, an airborne necrotrophic spore-producing ascomycete; *Sr*, a soilborne necrotrophic basidiomycete; and *Fol*, a soilborne hemibiotrophic ascomycete. Our results demonstrate that CK exhibits inhibitory activity against *Bc*, *Sr*, and, to a lesser degree, *Fol, in vitro*.

We found that CK treatment strongly inhibits *Bc* and *Sr* and inhibits *Fol* more weakly (Fig. 1a to e). CK inhibited the growth of *Bc* and *Sr* in a dose-dependent manner, achieving 40 to 60% growth inhibition of *Sr* and *Bc*, respectively, at 100 μM in solid media and ∼35% inhibition of *Bc* at 50 μM in liquid culture. Interestingly, various levels of inhibitory activity were observed for CK in many phytopathogenic fungi (Fig. S2). We found that CK has a stronger effect on *Bc* when the fungus is grown in liquid media (Fig. 1), achieving significant inhibition with 100 nM CK, perhaps due to higher penetration. Note that water content in tomato leaves is approximately 80% (63). We measured 200 to 400 nM *t*Z in tomato leaves (Fig. S3), suggesting that the concentration of CK within these leaves may have been sufficient to inhibit *Bc* during pathogenesis. Furthermore, we observed an increase in CK content 48 h after *Bc* infection (Fig. S3). Similar results were previously obtained with *Arabidopsis* (25), and we previously reported activation of the CK machinery upon exposure to *Bc* (23). This strengthens the notion that the plant may be using its CK as a defensive molecule to combat pathogen attack.

Examination of possible effects of CK on *Bc* development revealed that CK attenuates *Bc* sporulation, spore germination, and germ tube elongation (Fig. 2). Interestingly, genes involved in the regulation of virulence displayed significant downregulation by CK (Fig. 4c). This suggests that interference with the expression of virulence genes may partly contribute to the inhibitory effects of CK against *Bc*. Inhibition of hyphal growth and spore germination, together with downregulation of virulence genes in *Bc*, could account for disease attenuation in tomato. This mechanism could complement the plant-induced resistance mechanisms being activated by CK, which, as reported by us and others (20, 23, 64), induces plant immunity even in the absence of a pathogen. Our use of three different pathogens—two that produce spores (*Bc* and *Fol*) and one that does not (*Sr*), and two that infect via the soil (*Fol* and *Sr*) versus one that infects via the aerial plant parts (*Bc*)—indicates that CK-mediated disease reduction is not specific to any one lifestyle or infection mode, perhaps also pointing to a plant immunity component operating in parallel to any direct effects on the pathogen.

Damage to cell membrane integrity in fungi usually leads to the release of nucleic acids and proteins (65–67). CK showed no effect on leakage of nucleic acids and proteins from *Bc*, and no change was observed in medium conductivity of *Bc* after CK treatment, indicating that cell membrane permeability remained unchanged (Fig. 3). Taken together, these results suggest that CK is not toxic to fungi. CK can thus be defined as possessing fungistatic, and not fungicidal, activity, at least at concentrations up to 100 μM.

To understand the mode of action by which CK inhibited the growth of *Bc*, we conducted transcriptome profiling on *Bc* with and without CK. Transcriptome sequencing (RNA-seq) data suggested that CK downregulates the cell cycle and affects cytoskeleton and trafficking processes in *Bc* (Fig. 4). Thus, we examined cell morphology and DNA replication after CK application. CK strongly reduced cell area, distance between septa, and likely cell division. Cell division and septum formation are dependent on the signals generated during cell extension and growth, and on nuclear division. Reduced cell growth and elongation effected by CK treatment correlate with the lesser number of septa in the treated cells. Hoechst staining revealed that there was less DNA in CK-treated cells, likely due to inhibition of DNA replication processes and cell division, as observed in the transcriptome. Further supporting this notion is the fact that although cell sizes and DNA amounts were strongly reduced in the presence of CK, the ratio of DNA per septum remained unchanged by CK treatment (Fig. 5). Benomyl treatment, which is known to arrest the cell cycle (51), caused decreases in cell sizes that were similar to those effected by CK. The activity of benomyl and CK was additive when they were applied in sublethal concentrations (Fig. S5B). Inhibition of septum formation coupled with reduced DNA amounts and activity similar to benomyl, in light of the transcriptomic data, strongly supports the notion that CK inhibits cell division in fungal cells.

Also evident from the transcriptomic data was the effect of CK on the cytoskeleton and endocytic processes. Indeed, we found that CK caused mislocalization of actin at the growing tip of hyphae (Fig. 6), likely explaining, at least in part, the reduced hyphal growth observed (Fig. 1). CK inhibited the amount of endocytic vesicles in *Bc* (Fig. 7), S. cerevisiae (Fig. 8), and S. pombe (Fig. S6B), indicating that it likely has an impact on membrane function and/or fission of vesicles. CK itself may also enter fungal cells via endocytosis (68). Examining combined effects of CK and cytoskeleton inhibitors (Fig. S5B) demonstrated that they can have additive effects when used at sublethal concentrations, suggesting that they may partially act on similar targets within the cytoskeleton, though CK likely exerts its effect indirectly through additional target genes that remain to be identified.

Growth requires changes to both the composition and the orientation of the cytoskeleton (69). Cell elongation, in particular hyphal elongation, requires continuous addition of new plasma membrane, proteins, and cell wall material at the hyphal tip. Cellular trafficking and endo/exocytosis, which depend both on an intact cytoskeleton and on endocytic compartments, regulate the amount of membrane transferred toward this cellular growth, tightly controlling the amount of cellular material required for plasma membrane extension (44). Mislocalization of actin and inhibition of endocytosis by CK explain the reduced elongation and growth of *Bc* and yeast cells. Fission yeasts may be more reliant on cellular trafficking for rapid cell elongation than budding yeasts, and their growth is more similar to the growth of fungi than that of budding yeasts, possibly also explaining why fission yeast was more strongly inhibited by CK than budding yeast.

Previous work highlighted the importance of the actin cytoskeleton in *Bc* growth and virulence. Intact F-actin was found to be required for hyphal growth, morphogenesis, and virulence, which were all impaired in F-actin capping protein deletion mutants (70). Deletion of the *Bc* actin gene *bcactA* was also found to reduce growth and sporulation, and to lower virulence (71). Interestingly, the authors in that work found that *bcactA* regulates the—likely vesicular—secretion of extracellular virulence factors. These results further confirm the expected importance of the cytoskeleton in growth and virulence, supporting the idea that these processes are “targeted” by CK in the context of plant-pathogen interactions, with CK serving as an “antivirulence” molecule for the benefit of the plant.

We and others have previously demonstrated that CK serves as a cue to activate defense responses, and that plants activate CK signaling upon pathogen attack (20, 23, 64). *Bc* infection affected the levels of *t*Z in tomato leaves, causing a reduction after 24 h (23) and a subsequent increase after 48 h (Fig. S3b). A possibility arising from our work is that when plants sense the presence of phytopathogenic fungi, an additional reason for the activation of CK pathways is to promote CK biosynthesis, thereby inhibiting the growth of the potential fungal attacker. We have shown here that CK inhibits growth in different types of fungi from different classes (basidiomycetes and ascomycetes) and lifestyles (soilborne, airborne, hemibiotroph, and necrotroph) and yeasts (Fig. 1, 2, 5, and 8 and Fig. S1), and inhibits cellular trafficking in *Bc*, S. cerevisiae, and S. pombe (Fig. 7 and 8 and Fig. S5A and S6B). Genetically attenuating cellular trafficking in S. cerevisiae (Fig. 9) resulted in rescue of CK-mediated growth inhibition. It would seem that in employing CK as a fungal pathogen inhibitor, plants have targeted evolutionarily conserved processes fundamental to growth, such that this inhibition is preserved all the way to budding yeast.

It has been previously suggested that certain classes of fungi possess CK receptors to be able to “sense plants,” a trait posited to have been required for land colonization by fungi (72). CK sensing receptors were also reported for plant-pathogenic bacteria (73). Though *B. cinerea* is often considered necrotrophic, there are reports of *Bc* and additional necrotrophic plant fungal pathogens coexisting with the plant for extended periods before causing diseases (29), and a biotrophic phase for *B. cinerea* has been described previously (74), suggesting that the ability of *B. cinerea* to sense the environment within the plant is important for its survival. This brings forth the possibility that phytopathogenic fungi can use CK sensing as an indicator of the plant status and possibly react accordingly. Interestingly, many fungal phytopathogens have genes that could produce CK (75, 76), and fungus-derived CK was reported to play a role in several plant-fungus interactions (77–80). However, in most cases, it is not known whether fungi produce CKs when in contact with the host plant, and thus, a universal role for fungal CK is not yet evident. Additional work is needed to elucidate this point.

Here, we have provided evidence for a new role for CK in plant-pathogen interactions. CK was known to induce plant immunity, and we show that it also directly inhibits fungal phytopathogens. It seems that CK plays a more central role in the evolutionary war against plant pathogens than previously thought. Plants appear to have a dual mechanism for employing CK, previously believed to be only a “developmental” hormone, when interacting with a fungal pathogen: (i) the plant senses a pathogen and activates its CK response, leading to immunity signaling, which culminates in increased immunity and systemic pathogen resistance, and (ii) the activation of CK response leads after 48 h to the generation of increased CK levels, which inhibit the growth and development of fungal pathogens by targeting their cell cycle, cytoskeleton, and trafficking machinery.

Our work uncovers a novel, remarkably conserved role for a primary plant growth hormone in fungal biology, suggesting that interactions between pathogen and host resulted in fascinating molecular adaptations on fundamental processes in eukaryotic biology. Future work will validate the genetic targets of CK in fungi and explore whether the fungistatic activity it possesses can be agriculturally adapted into the broad context of the roles of CK in plant life. In time, this may hold promise for the development of CKs as antifungal agents in specific cases.

## MATERIALS AND METHODS

### Pathogen growth conditions.

*Botrytis cinerea* (strain Bcl16), *Sclerotium rolfsii* (81), and Fusarium oxysporum f. sp. *lycopersici* (strain 4287) were cultivated on potato dextrose agar (PDA) at 22 ± 2°C for *B. cinerea*, 26 ± 2°C for *S. rolfsii*, and 28 ± 2°C for F. oxysporum for 5 to 7 days. Pathogen isolates were kindly gifted by Yigal Elad, David Ezra, and Shay Covo.

### Mycelial growth assays.

To study the effect of CK on mycelial growth of pathogenic fungi, 6-benzylaminopurine (6-BAP; Sigma-Aldrich), zeatin (Sigma-Aldrich), kinetin (Sigma-Aldrich), TDZ (Sigma-Aldrich), and adenine (Sigma-Aldrich) were dissolved in 10 mM NaOH and 1 M HCl and added to PDA media at a concentration gradient of 0, 1, 10, and 100 μM. Mock controls contained equal amounts of the solvent. *B. cinerea*, *S. rolfsii*, and F. oxysporum mycelial plugs (5 mm) taken ∼1 cm from the edge of a fresh plate were placed at the center of PDA plates and incubated under the above-mentioned growth conditions. For liquid growth assays, to measure the mycelium weight of *B. cinerea*, *Bc* was cultured in stationary liquid PDB media in the presence of 0, 100, and 500 nM and 1, 5, 10, and 50 μM concentrations of 6-BAP. After 72 h, the fungal mass was dried and the dry weight was measured.

Adenine is commonly used as a control due to its structural similarity to cyclic CKs (2). The effect of 6-BAP on the growth of all phytopathogenic fungi was studied similarly. Since 6-BAP treatment resulted in strong inhibition, and 6-BAP has increased stability over the other derivatives tested, following the testing of different CK compounds, we conducted all subsequent assays using 6-BAP.

### Cytokinin level measurement.

To analyze the active CK levels in mature tomato (cv. M82) leaves, phytohormone extraction was performed as follows. Leaf tissue was ground in liquid nitrogen, and 200 to 450 mg of fresh ground tissue powder from 4-week-old (active compound quantification) or 6-week-old (for *t*Z quantification, mock or *Bc* infected) was transferred to a 2-ml tube containing 1 ml of extraction solvent (79% isopropyl alcohol, 20% methanol, and 1% acetic acid) mixture supplemented with 20 ng of deuterium-labeled internal standards (IS; Olomouc, Czech Republic). The tubes were incubated at 4°C for 1 h on a shaker and centrifuged at 14,000 × *g* for 15 min at 4°C. The supernatant was transferred to 2-ml tubes, 500 μl of extraction solvent was added to the pellet, and the extraction steps were repeated twice. The combined extracts were evaporated using SpeedVac at room temperature, and dried samples were dissolved in 200 μl of 50% methanol and further filtered with a 0.22-μm cellulose syringe filter. A volume of 5 to 10 μl was injected for each analysis. LC-tandem mass spectrometry (MS/MS) analyses were conducted using an ultrahigh-performance liquid chromatograph (UPLC)-triple quadrupole MS (TQMS; Waters; Xevo). Separation was performed on Waters Acuity UPLC BEH Ethylene Bridged Hybrid C_18_ 1.7-μm, 2.1- by 100-mm column with a VanGuard precolumn (BEH C_18_, 1.7 μm, 2.1 by 5 mm). The mobile phase consisted of water (phase A) and acetonitrile (phase B), both containing 0.1% formic acid in the gradient elution mode. The flow rate was 0.3 ml/min, and the column temperature was kept at 35°C. Acquisition of LC-MS data was performed using Mass Lynx V4.1 software (Waters). Quantification was done using isotope-labeled IS. Solvent gradients and MS-MS parameters are detailed in Table S1.

10.1128/mBio.03068-20.8 TABLE S1 Solvent gradients and MS-MS parameters for CK quantification. Download Table S1, PDF file, 0.08 MB. Copyright © 2021 Gupta et al. 2021 Gupta et al. https://creativecommons.org/licenses/by/4.0/ This content is distributed under the terms of the Creative Commons Attribution 4.0 International license.

### Plant pathogenesis assays.

*S. lycopersicum* cv. M82 seeds were sown after surface sterilization (with 1.5% NaOCl for 5 min, followed by three rinses with sterile water) in a tray containing potting mixture. Five days after germination, tomato seedlings were transplanted to 4.5-liter pots containing green quality soil mix (Even-ari, Ashdod, Israel). Plants were kept in a greenhouse at 25 ± 2°C and a 16-h photoperiod.

To study the effect of CK on *Bc* pathogenicity, *Bc* was grown on PDA in the dark at 22 ± 2.7°C; 10-day-old plates were given daylight for 6 h and then returned to the dark for sporulation. Spores were harvested in 1 mg ml^−1^ of glucose and 1 mg ml^−1^ of K_2_HPO_4_ and filtered through sterile cheesecloth. The spore concentration was adjusted to 10^6^ spores ml^−1^ after quantification under a light microscope using a Neubauer chamber.

A solution of 6-BAP (100 μM, diluted in 10 mM NaOH) with Tween 20 (100 μl liter^−1^) was sprayed onto 4-week-old plants 24 h before pathogen inoculation. Mock-treated plants were sprayed with a solution of 10 mM NaOH and 100 μl liter^−1^ of Tween 20. The second left-hand lateral leaflet from the fourth leaf of each plant was inoculated with two droplets of 10-μl spore suspension (82). Disease, as expressed by lesion area of the necrotic tissue, was measured after 5 days of pathogen inoculation using ImageJ software.

*Fol* culture was grown in KNO_3_ medium (for 1 liter: 1.36 g of yeast nitrogen base, 24 g of sucrose, and 100 mM KNO_3_) at 28°C for 5 days (83). Spores were harvested from the liquid culture and adjusted to 10^6^ spores ml^−1^ for inoculation (84). Two-week-old tomato plants were treated with 100 μM 6-BAP through foliar spray and inoculated using the root dip method (85, 86) with the pathogen spore suspension 24 h after 6-BAP application. As a control, noninoculated tomato plants were used. The disease index (DI) was calculated after 3 weeks of pathogen infection using a scale from 0 to 5 as follows: 0, no symptoms; 1, ≥2% (healthy plant); 2, 3 to 30% (slight disease); 3, 31 to 60% (moderate disease); 4, 61 to 90% (severe disease); and 5, ≤91% (dead plant).

For *Sr*, 2-week-old tomato plants were soil drenched with 100 μM 6-BAP three times on alternate days, for a total of 3 treatments, prior to infection. Mock-treated plants were drenched with a solution of 10 mM NaOH and 100 μl liter^−1^ of Tween 20. At 24 h after the third treatment, 3 or 4 sclerotia of *Sr* were placed on the soil, ∼2 cm from the plant stem. Mass culture of sclerotia of *Sr* was done on PDA media by growing the fungus for 14 days in the dark at 28°C and then transferring it to direct sunlight until sclerotia formed. Sclerotia were collected with a sterilized paintbrush and stored in 4°C until use. The first symptoms appeared 1 week after inoculation and ultimately developed into severe rot and wilted the plants. The DI was calculated by using the above-mentioned 0-to-5 scale.

### *B. cinerea* sporulation and spore germination measurement.

To study the effect of sporulation, a *Bc* hyphal plug (5 mm) from a 5-day-old PDA plate cultured as described above was placed at the center of PDA plates containing 0, 0.1, and 100 μM 6-BAP for 5 days at 22 ± 2°C in the dark. Plates were then transferred to sunlight for 6 h and returned to the dark. Spore production was assessed 4 days following light exposure. The isolate Bcl-16 sporulates well on PDA plates. Since *Bc* spores are black while the mycelium is white, plates were analyzed for black color, i.e., spore formation, in four independent experiments of at least 3 replicates. For spore germination, *B. cinerea* spores at a concentration of 10^6^ spores ml^−1^ were incubated in potato dextrose broth (PDB) containing 0 and 100 μM 6-BAP at 22 ± 2°C for 8 h. Thereafter, conidia were washed twice in sterile water (1 ml) and centrifuged at 12,000 rpm for 5 min, and the pellet was resuspended in 100 μl of sterile water. A 10-μl sample was analyzed under the microscope. The percent sporulation, spore germination, and length of germ tubes were measured using ImageJ software, the count tool for spore germination, and the measure tool for germ tube length.

### Measurement of cellular leakage and electrolytes.

*Bc* spores were grown in PDB supplemented with 6-BAP (0 and 100 μM) and incubated on a rotary shaker at 180 rpm for 24 h at 22°C. The mycelia were subsequently centrifuged and the aqueous supernatants were used for measurement of the leakage of nucleic acids and proteins. The concentration of the proteins released from the cytoplasm was measured using the Bradford method (87). The absorbance of the supernatant was measured at 595 nm using a UV-visible (UV-Vis) spectrophotometer, and leakage of proteins was quantified according to the method of Bradford (87). The release of nucleic acids in various treatments was measured by detecting the optical density at 260 nm. 6-BAP resembles a nucleotide, as it has an absorbance spectrum at 260 nm. Electrical conductivity was measured using a conductivity meter (Eutech; instrument con510) after 24 h.

### RNA extraction, quality control, and RNA sequencing.

Total RNA was extracted from liquid *B. cinerea* cultures grown for 48 h in 1/2 PDB with the addition of tobacco seedlings 5 days postgermination (150 seedlings/50 ml of medium), mock treated or supplemented with 25 μM 6-BAP, 50 mg of fungal mass per sample, using the Norgen total RNA purification kit (Norgen Biotek Corp.) according to the manufacturer’s instructions. RNA yield and purity were measured by NanoDrop (ND-1000 spectrophotometer; NanoDrop, Wilmington, DE). RNA integrity was assessed by electrophoresis on a 1.5% agarose gel. RNA quality was validated for by running on a Bioanalyzer 2200 TapeStation (Agilent Technologies, CA). cDNA libraries were prepared for sequencing using the TruSeq RNA kit (Illumina, San Diego, CA). Libraries were evaluated with Qbit and TapeStation (Agilent Technologies). Sequencing libraries were constructed with barcodes for sample multiplexing. Pooled libraries of the 6 samples were sequenced on one lane of an Illumina HiSeq 2500 instrument using a 60-bp single-end RNA-Seq protocol to obtain ∼20 million reads per sample. Sequencing was performed at the Weizmann Institute of Science, Israel.

### Transcriptome analysis.

Raw reads were subjected to a filtering and cleaning procedure. The Trimmomatic tool was used to filter out adapter sequences, remove low-quality sequences by scanning a 4-base wide sliding window, cutting when the average quality per base drops below <15 and finally, removal of reads shorter than 36 bases (88). Clean reads were mapped to the reference genomes of *Botrytis cinerea* B05.10 (RefSeq assembly accession no. GCF_000143535.2) (89) using STAR software (90). Gene abundance estimation was performed using Cufflinks version 2.2 (91) combined with gene annotations from GenBank. Heat map visualization was performed using R Bioconductor (92). Gene expression values were computed as fragments per kilobase per million (FPKM). Differential expression analysis was completed using the DESeq2 R package (93). Genes with an adjusted *P* value of no more than 0.05 were considered differentially expressed. PCA was done using the R function prcomp. We submitted the raw sequencing data generated in this study to NCBI under BioProject accession number PRJNA718329.

The gene sequences were used as a query term for a search of the NCBI nonredundant (nr) protein database that was carried out with the DIAMOND program (94). The search results were imported into Blast2GO version 4.0 (95) for Gene Ontology (GO) assignments. Gene Ontology enrichment analysis was carried out using the Blast2GO program based on Fisher’s exact test with multiple testing correction of false-discovery rate (FDR). The KOBAS 3.0 tool (http://kobas.cbi.pku.edu.cn/kobas3/?t=1) (96) was used to detect the statistical enrichment of differential expression genes in KEGG pathway and GO.

### Cell elongation and DNA content.

To examine the cell morphology of *Bc* spores treated with 6-BAP, 1 g liter^−1^ of calcofluor white M2R (Sigma-Aldrich) was used. Cells were cultured in PDB with or without 6-BAP (100 μM) at 22°C for 16 h. The cells were then collected and stained with calcofluor white M2R at room temperature for 15 min. Additionally, for septum counting after 8 h of CK cocultivation, growing hyphae from germinating spores were similarly stained and visualized under a light microscope.

For DNA replication, a stock solution of 1.5 mg ml^−1^ of Hoechst 33342 (Sigma-Aldrich) dye was prepared in water. The stock solution was further diluted 1:100 using water and applied to *Bc* spores cultured in PDB with or without 6-BAP (100 μM) for 16 h at 22°C. The *Botrytis* samples stained with Hoechst were plated on microscope slides, and the slides were incubated in the dark for 1 h in a humid chamber at room temperature before microscopic visualization (51). Nuclei and septa were visualized with either a light or a fluorescence Olympus microscope, using the 4′,6-diamidino-2-phenylindole (DAPI) channel, and the images were analyzed using Fiji-ImageJ. Cell area, as well as linear distance between septa, was measured in individual hyphae. At least 120 hyphae from at least 24 images of each treatment captured in 6 separate experiments were used for cell area measurements, and at least 30 hyphae from at least 10 images of each treatment captured in 3 separate experiments were used for measuring the distance between septa. Cell size and length (septal distance) of individual hyphal cells were measured using the area measurement tool for cell size and the line measurement tool for the distance between septa. Septa were also counted using the counter tool. DNA staining was assessed using the mean intensity measurement tool.

### *B. cinerea* qRT-PCR.

To examine the effect of CK on cytoskeleton genes, we grew *Bc* from spores in PDB with the addition of 100 μM CK (6-BAP) or without (mock) in a rotary shaker at 180 rpm and 22 ± 2°C for 48 h. Total RNA was isolated from equal fungal masses of mock- and CK-treated samples, using Tri reagent (Sigma-Aldrich) according to the manufacturer’s instructions. RNA (3 μg) was used to prepare cDNA using reverse transcriptase (Promega, USA) and oligo(dT)15. qRT-PCR was performed on a Step One Plus real-time PCR system (Thermo Fisher, Waltham, MA) with the Power SYBR green master mix protocol (Life Technologies, Thermo Fisher, USA). The primer sequences for each gene, and primer pair efficiencies, are detailed in Table S2A. A geometric mean of the expression values of the three housekeeping genes for ubiquitin-conjugating enzyme E2 (97), iron transport multicopper oxidase, and adenosine deaminase (98) was used for normalization of gene expression levels. Relative gene expression levels were calculated using the threshold cycle (2^−ΔΔ^*^CT^*) method (99). At least six independent biological replicates were used for analysis.

10.1128/mBio.03068-20.9 TABLE S2 (A) Primers used in RT-qPCR. (B) Oligonucleotides used for generating and validating Saccharomyces cerevisiae mutant strains. (C) Oligonucleotides used for generating and validating *Botrytis cinerea* mutant strains. Download Table S2, PDF file, 0.1 MB. Copyright © 2021 Gupta et al. 2021 Gupta et al. https://creativecommons.org/licenses/by/4.0/ This content is distributed under the terms of the Creative Commons Attribution 4.0 International license.

### *B. cinerea* transformation.

For generation of *B. cinerea* mutants expressing Lifeact-GFP, we prepared a fusion construct to target replacement of the nitrate reductase (*bcniaD*) gene. For generation of constructs expressing Lifeact-GFP at the *bcniaD* locus, we used the plasmid pNDH-OLGG as a template (45). The vector contains 5′ and 3′ flanking sequences of *bcniaD*, a resistance cassette mediating resistance to hygromycin, and the filamentous actin (F-actin) imaging probe Lifeact fused to GFP. The expression cassette carrying the hygromycin resistance gene and the *bcniaD* flanking sequence was amplified using primers GA 34F/34R (Table S2C). The PCR-amplified expression cassette was used to transform *B. cinerea* using polyethylene glycol (PEG)-mediated transformation (100). A 0.125% lysing enzyme from Trichoderma harzianum (Sigma-Aldrich, Germany) was used for protoplast generation. Following PEG-mediated transformation, protoplasts were plated on SH medium containing sucrose, Tris-Cl, (NH_4_)_2_HPO_4_, and 35 μg/ml of hygromycin B (Sigma-Aldrich). The colonies that grew after 2 days of incubation were transferred to PDA-hygromycin medium, and conidia were spread again on selection plates to obtain a monoconidial culture. Since strong expression of Lifeact-GFP mediated by the oliC promoter results in toxicity in homokaryotic *B. cinerea* strains, fungal transformants were visualized under a confocal microscope and screened with primers GA 34F/34R and GA 31F/31R (Table S2C). Confirmed transformants were stored at −80°C and used for further experiments.

### *B. cinerea* endocytosis.

To measure endocytosis in *Bc* hyphae, the culture was grown in PDB with or without 6-BAP (100 nM and 100 μM), benomyl (Sigma; catalog no. 17804-35-2), or latrunculin B (Sigma; catalog no. 76343-94-7) for 16 h at 22°C, after which the cells were collected and stained with 5 μM FM4-64 (5 min on ice) on a glass coverslip. We acquired confocal microscopy images using a Zeiss LSM780 confocal microscope equipped with a 63×/1.15 Corr objective. FM4-64 images were acquired with a 514-nm excitation laser (4% power), with the emission collected in the range of 592 to 768 nm. Images of 8 bits and 1,024 by 1,024 pixels were acquired using a pixel dwell time of 1.27 μs, pixel averaging of 4, and pinhole of 1 Airy unit (1.3 μm). Image analysis (18 to 24 images per treatment collected in three independent experiments) was conducted with Fiji‐ImageJ using the raw images and the three-dimensional (3D) object counter tool and measurement analysis tool (101). Endosome density and size were calculated automatically by the software tool considering a 1.3-μm depth, based on a single optical section.

Corrected total fluorescence (CTF) was measured for the spitzenkorper (∼6 μm^2^ at the growing hypha tip) and actin content in the cell by using the integrated density (IntDen) function in ImageJ and subtracting the background mean fluorescence times the selected area from the measured integrated density, i.e., CTF = integrated density – (area of selected cell × mean fluorescence of background readings).

To examine the additive effects of CK and cytoskeleton inhibitors (Ben and LatB) on fungal growth, we cultured *Bc* on PDA plates in the presence of concentrations of CK (6-BAP; 5 μM and 100 μM) and Ben (10, 100, and 500 nM and 1 μM) or LatB (1, 10, 100, and 500 nM) for 48 h at 22°C.

### Budding (Saccharomyces cerevisiae) and fission (Schizosaccharomyces pombe) yeast growth.

Wild-type haploid yeast strains were grown overnight at 30°C. S. cerevisiae cells were grown in synthetic defined (SD) medium and S. pombe cells were grown in Edinburgh minimal medium (EMM), without (mock) or with the addition of indicated concentrations of CK (6-BAP). S. cerevisiae and S. pombe were diluted to an OD_600_ of 0.01. Cells (200 μl) were plated in 96-well plates and incubated at 30°C for 25 h (S. cerevisiae) or 45 h (S. pombe), with continuous shaking. OD_600_ was measured using a Tecan SPARK 10M plate reader. The experiment was repeated three times, with similar results. WT strains of S. cerevisiae and S. pombe were kind gifts from Martin Kupiec and Ronit Weisman.

### Budding (S. cerevisiae) and fission (S. pombe) yeast endocytosis.

Saccharomyces cerevisiae was grown overnight in YPD (Yeast extract, peptone, dextrose) media, and then the culture was diluted (OD_600_ = 0.2) and incubated for 6 h in YPD (mock) or YPD supplemented with 300 μM 6-BAP. S. pombe was grown overnight in YE yeast extract medium. The cultures were then diluted (OD_600_ = 0.2) and incubated for 6 h in YE media (mock) or media supplemented with 100 μM 6-BAP. Cell cultures were collected by centrifugation at 5,000 rpm for 4 min and resuspended in fresh growth medium. FM4-64 staining was performed as described previously (57). Cells were incubated with 24 μM FM4-64 (Invitrogen) for 30 min at 4°C. Subsequently, the FM4-64-containing medium was replaced with fresh medium, and cultures were incubated for 15 min at 28°C. To observe FM4-64 distribution, 5-μl volumes of the suspensions were placed on a slide and live confocal imaging was performed. Confocal microscopy images were acquired using a Zeiss LSM780 confocal microscope equipped with objective LD C-Apochromat 63×/1.15 Corr. Acquisition settings were designed using an excitation laser wavelength of 514 nm (4% power). The emission was then collected in the range of 592 to 768 nm. Images of 8 bits and 1,024 by 1,024 pixels were acquired using a pixel dwell time of 1.27 μs, pixel averaging of 4, and pinhole of 1 Airy unit. Bright field was acquired using the T-PMT (transmitted light detector). Image analysis was performed using Fiji-ImageJ with the raw images (101), endosome count and size measurements were performed with the 3D object counter tool, and pixel intensity was measured using the measurement analysis tool. At least 160 individual cells obtained from 15 to 21 images per treatment were analyzed. Total pixel intensity was measured in a defined region of interest (ROI) in each cell that excludes the plasma membrane. This ROI was subsequently used to quantify endosomal compartments in each cell.

### Construction of budding yeast mutant strains.

The *YPT31*, *SSA1*, *VPS1*, and *SPO14* genes were disrupted in wild-type yeast strain BY4741 via homologous recombination using PCR fragments amplified from plasmid pFA6a-KanMX6 as a template with suitable primers. Gene replacement was validated by PCR with suitable primers. Primer sequences are provided in Table S2B.

### Data analysis.

Data are presented as the averages ± the standard errors of the means (SEM), or minimum to maximum values. For Gaussian-distributed samples, we analyzed the statistical significance of differences between two groups using a two-tailed *t* test, with additional *post hoc* correction where appropriate, such as FDR calculation with Holm-Sidak correction in growth curve assays and Welch’s correction for *t* tests between samples with unequal variance. We analyzed the statistical significance of differences among three or more groups using analysis of variance (ANOVA). Regular ANOVA was used for groups with equal variances, and Welch’s ANOVA was used for groups with unequal variances. Significance in differences between the means of different samples in a group of 3 or more samples was assessed using a *post hoc* test. The Tukey *post hoc* test was used for samples with equal variances, when the mean of each sample was compared to the mean of every other sample. The Bonferroni *post hoc* test was used for samples with equal variances, when the mean of each sample was compared to the mean of a control sample. The Dunnett *post hoc* test was used for samples with unequal variances. For samples with non-Gaussian distribution, we analyzed the statistical significance of differences between two groups using a Mann-Whitney U test, and the statistical significance of differences among three or more groups using Kruskal-Wallis ANOVA, with Dunn’s multiple-comparison *post hoc* test as indicated. Gaussian distribution or lack thereof was determined using the Shapiro-Wilk test for normality. Statistical analyses were conducted using Prism8^T^.

### Data availability.

The data supporting the findings of this study are available above and within the supplemental material. Raw data are available from the corresponding author upon reasonable request. The raw data generated in the transcriptomic analyses are deposited in NCBI under BioProject accession number PRJNA718329.
