# Erratum for Gupta et al., “Cytokinin Inhibits Fungal Development and Virulence by Targeting the Cytoskeleton and Cellular Trafficking”

**DOI:** 10.1128/mbio.00305-22

**Published:** 2022-03-14

**Authors:** Rupali Gupta, Gautam Anand, Lorena Pizarro, Dana Laor Bar-Yosef, Neta Kovetz, Noa Sela, Tal Yehuda, Ehud Gazit, Maya Bar

**Affiliations:** a Department of Plant Pathology and Weed Research, ARO, Volcani Institute, Rishon LeZion, Israel; b Institute of Agri-food, Animal and Environmental Sciences, Universidad de O’Higgins, Rancagua, Chile; c The Shmunis School of Biomedicine and Cancer Research, Tel Aviv University, Tel Aviv, Israel

## ERRATUM

Volume 12, no. 5, e03068-20, 2021, https://doi.org/10.1128/mBio.03068-20. Dana Laor Bar-Yosef’s surname was truncated in the original article. The byline should appear as shown above. The article has been corrected online.

