# Supplementary material for: Cytokinin Inhibits Fungal Development and Virulence by Targeting the Cytoskeleton and Cellular Trafficking
Source: mBio. 2021 Oct 19;12(5):e03068-20. doi: 10.1128/mBio.03068-20 (PMC8524340; doi:10.1128/mBio.03068-20)
Supplement: FIG S1 [file mbio.03068-20-sf001.pdf]

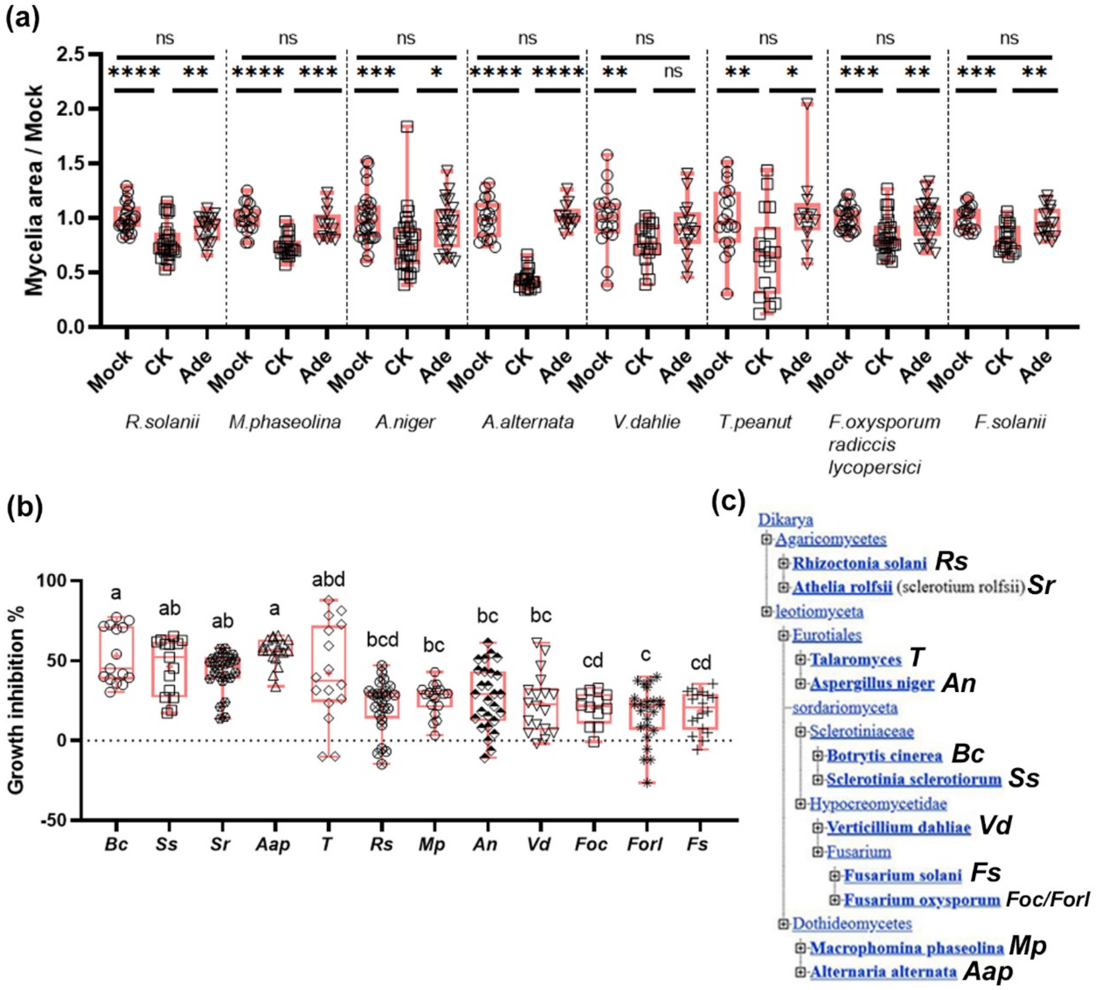

Fig. S1. Cytokinin inhibits fungal growth.

(a) Growth of different fungi cultured on potato dextrose agar (PDA) plates in the presence of 100 µM CK (6-Benzylaminopurine), or the control Adenine (Ade). Quantification of results from 4-6 biological repeats ±SE, N>12. Asterisks indicate significance in a Mann-Whitney U test, **p*<0.05, ***p*<0.01, ****p*<0.001; *****p*<0.0001; ns (non-significant). **(b)** Comparison of the inhibition level of CK for different fungi. Letters indicate significance in a Kruskal-Wallis ANOVA with Dunn's post hoc test, p<0.04. The phylogeny is detailed in **(c). a-b:** Box plots with all individual values shown, line indicates median.
