# Supplementary material for: Cytokinin Inhibits Fungal Development and Virulence by Targeting the Cytoskeleton and Cellular Trafficking
Source: mBio. 2021 Oct 19;12(5):e03068-20. doi: 10.1128/mBio.03068-20 (PMC8524340; doi:10.1128/mBio.03068-20)
Supplement: FIG S2 [file mbio.03068-20-sf002.pdf]

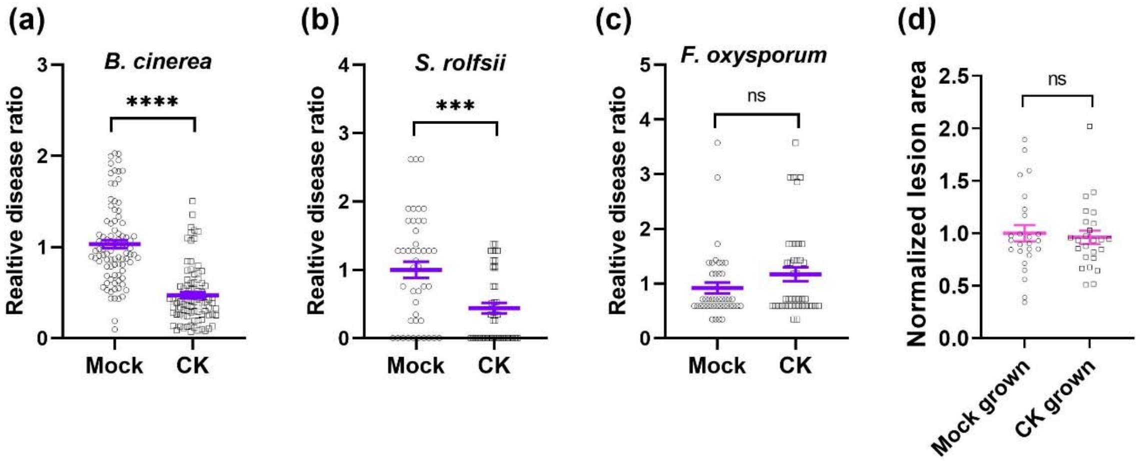

Fig. S2. Cytokinin inhibits on-plant pathogenesis of *B. cinerea* and *S. rolfsii*, but not *F. oxysporum.*

S. lycopersicum cv M82 leaves were treated with 100 µM CK (6-Benzylaminopurine) and inoculated with indicated pathogens 24 hours later. Plant disease was quantified as indicated in the material and methods section, disease in the mock treatment was set to 1. Graphs represent results from 4-6 biological repeats ±SE, N>40. Asterisks represent significant difference among means in a Mann-Whitney U test, ***p<0.001, ****p<0.0001, ns-non-significant. All individual values graphed, purple/pink bar represents mean ±SE.
