# Supplementary material for: Cytokinin Inhibits Fungal Development and Virulence by Targeting the Cytoskeleton and Cellular Trafficking
Source: mBio. 2021 Oct 19;12(5):e03068-20. doi: 10.1128/mBio.03068-20 (PMC8524340; doi:10.1128/mBio.03068-20)
Supplement: FIG S3 [file mbio.03068-20-sf003.pdf]

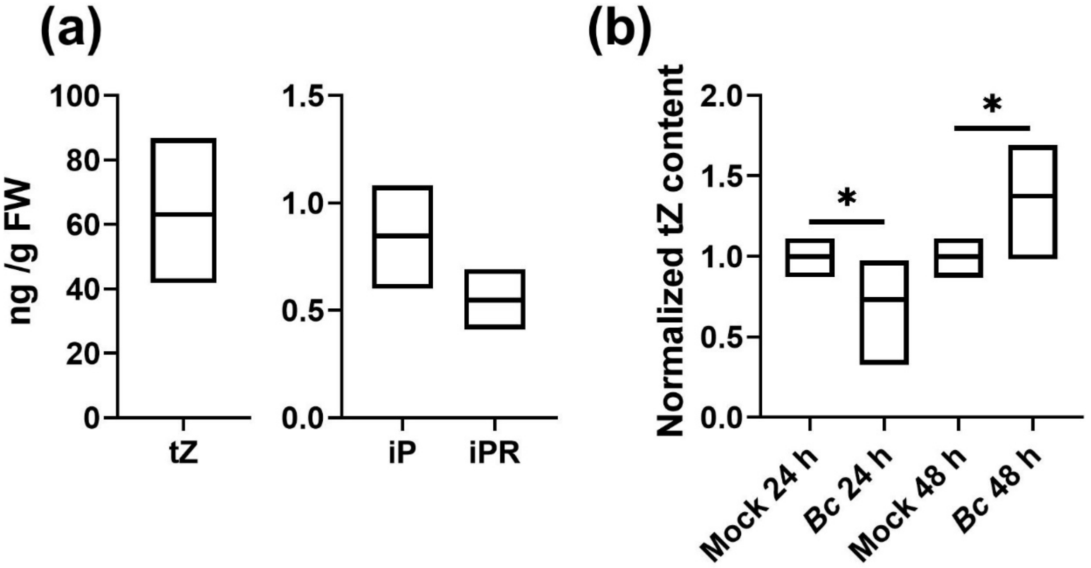

Fig. S3. Quantification of active CKs in tomato leaves using LC–MS-MS.

Fresh ground tissue powder was iso-propanol/methanol extracted in the presence of deuterium-labelled internal standards. LC–MS-MS analyses were conducted using a UPLC-Triple Quadrupole MS (WatersXevo TQMS). Acquisition of LC–MS data was performed using Mass Lynx V4.1 software (Waters). (**a**) Quantification of CKs (tZ, transzeatin, iP, isopentenyladenine and iPR, iP riboside) was done on 4 week old *S. lycopersicum* cv. M82 leaves using isotope-labeled internal standards (IS), as described in the methods section. Quantification of results from 3 biological replicas. Bars represent minimum-maximum value range, with line indicating mean. (**b**) Quantification of tZ in Mock and *Bc* inoculated plants, 24 h and 48 h after *B. cinerea* inoculation, on 6 week old *S. lycpersicum* cv. M82 leaves. Quantification of results from 4 biological replicas. Bars represent minimum-maximum value range, with line indicating mean. Asterisk indicates significance from Mock in a two-tailed t-test, $*p<0.05$.
