# Supplementary material for: Cytokinin Inhibits Fungal Development and Virulence by Targeting the Cytoskeleton and Cellular Trafficking
Source: mBio. 2021 Oct 19;12(5):e03068-20. doi: 10.1128/mBio.03068-20 (PMC8524340; doi:10.1128/mBio.03068-20)
Supplement: FIG S4 [file mbio.03068-20-sf004.pdf]

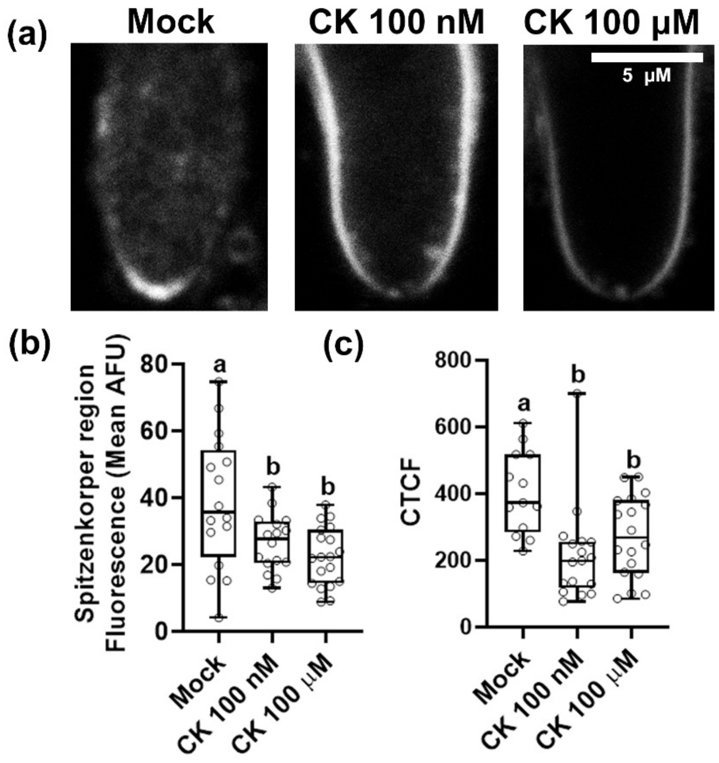

Fig. S4. Cytokinin affects the integrity of the Spitzenkorper.

B. cinerea (*Bc*) was cultured in PDB liquid broth in the presence of 100 nM or 100 µM CK (6-Benzylaminiopurine) for 16 hours. **(a)** FM-4-64 staining of the Spitzenkorper (Spk) region in *Bc* hyphae. **(b-c)** Quantification of the FM-4-64 mean fluorescence (b) and FM-4-64 corrected total cellular fluorescence (c) in the Spk region. Measurements were done using the measurement tool of Fiji. Quantification of results from 3 biological repeats. N>16, box-plot with all values displayed, box indicates inner-quartile ranges with line indicating median, whiskers indicate outer-quartile ranges. (b) Different letters indicate statistically significant differences among samples in one-way ANOVA with a Tukey post hoc test, $p< 0.031$, (c) Different letters indicate statistically significant differences among samples in Kruskal-Wallis ANOVA with Dunn's post hoc test, $p<0.034$.
