# Supplementary material for: Cytokinin Inhibits Fungal Development and Virulence by Targeting the Cytoskeleton and Cellular Trafficking
Source: mBio. 2021 Oct 19;12(5):e03068-20. doi: 10.1128/mBio.03068-20 (PMC8524340; doi:10.1128/mBio.03068-20)
Supplement: FIG S5 [file mbio.03068-20-sf005.pdf]

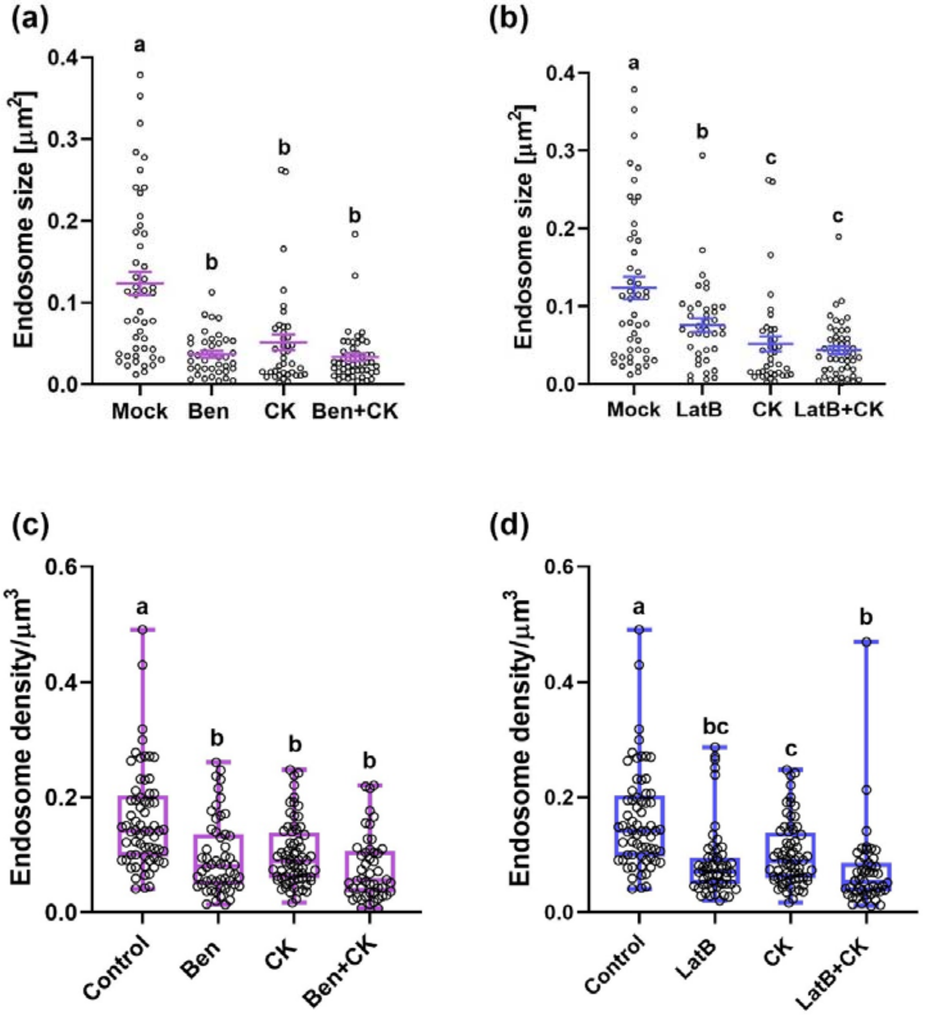

Fig. S5A. Inhibition of the cellular cytoskeleton affects *B. cinerea* cytokinin sensitivity-cellular trafficking

B. cinerea (*Bc*) was cultured in PDB liquid broth in the presence of 100 µM CK (6-Benzylaminiopurine) and/ or 1 µ M Benomyl (Ben; **a,c**) or Latrunculin B (LatB; **b,d**) for 8 hours. FM-4-64 endocytic vesicles were analyzed in growing hyphae. Measurements were done using the counting tool of Fiji. **(a,b)** Quantification of the average size of vesicles from 3 biological repeats, N>40 images, the average endosome size per image was used for the analysis. All points displayed, mean ±SE is indicated. **(c,d)** Quantification of the amount of endocytic vesicles from 5 biological repeats, N>50 images. Box-plots with all values displayed, box indicates inner-quartile ranges with line indicating median, whiskers indicate outer-quartile ranges. Different letters indicate significance between samples in a Kruskal-Wallis ANOVA with Dunn's post hoc test, a: p<0.0001; b: p<0.028; c: p<0.0002, d: p<0.002.

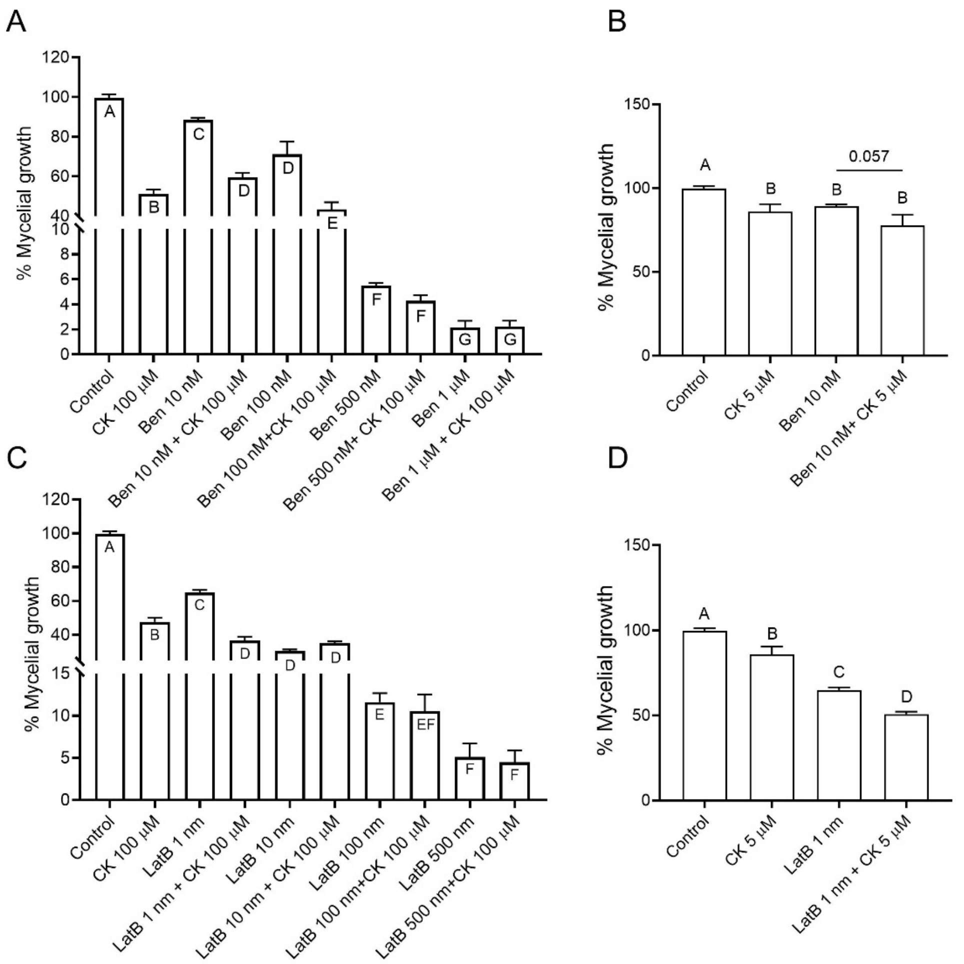

Fig. S5B. Additive effects of CK and Cytoskeleton inhibitors on fungal growth

(a-b) *B. cinerea* (*Bc*) was cultured on PDA plates in the presence of indicated concentrations of CK (6-Benzylaminiopurine) and Benomyl (Ben) for 48 hours.

(c-d) *B. cinerea* (*Bc*) was cultured on PDA plates in the presence of indicated concentrations of CK (6-Benzylaminiopurine) and Latrunculin B (LatB) for 48 hours.All graphs represents mean ±SE, N=6. Different letters indicate statistically significant differences between samples in a one-way ANOVA with a Tukey post hoc test, A: $p<0.03$; B: $p<0.048$; C: $p<0.032$; D: $p<0.0052$.
