# Supplementary material for: Cytokinin Inhibits Fungal Development and Virulence by Targeting the Cytoskeleton and Cellular Trafficking
Source: mBio. 2021 Oct 19;12(5):e03068-20. doi: 10.1128/mBio.03068-20 (PMC8524340; doi:10.1128/mBio.03068-20)
Supplement: FIG S6 [file mbio.03068-20-sf006.pdf]

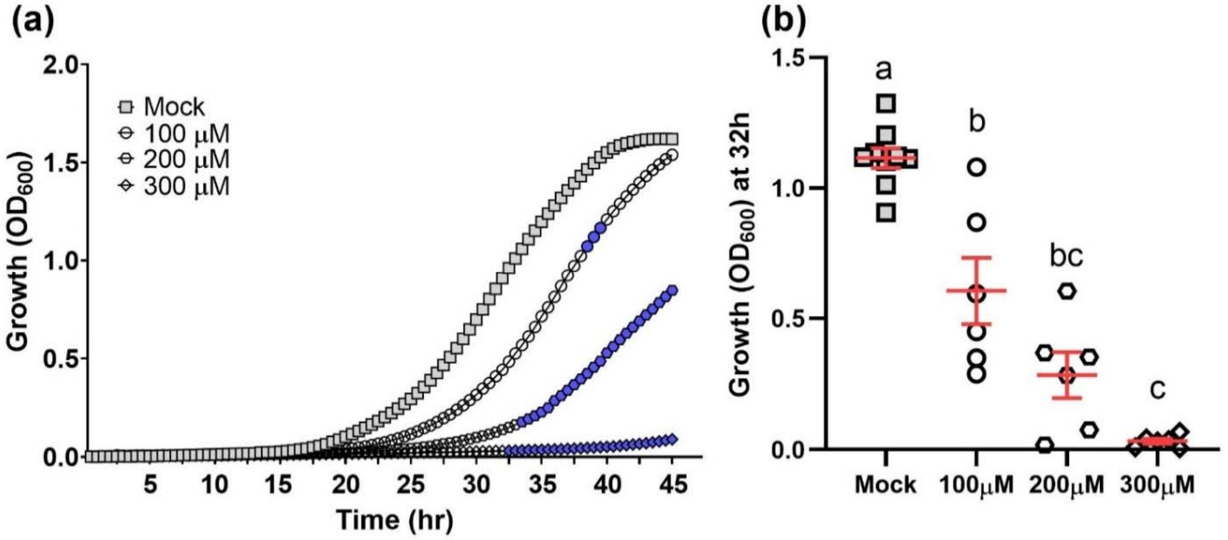

Fig. S6A. Cytokinin inhibits the growth of fission yeast.

(a) Wild-type *Schizosaccharomyces pombe* cells were grown over night at 30°C in minimal EMM medium, treated with either 10uM NaOH (Mock) or with the addition of indicated concentrations of CK (6-Benzylaminopurine). Cells were incubated at 30°C for 45h, with continuous shaking. Average growth per time point for three independent experiments is presented, N=6. Blue color represents statistical significance in a two-tailed t-test with Holm-Sidak correction, $p<0.05$. **(b)** Average growth (OD) at mid log phase (32h) in three independent experiments. Letters indicate significance in a one-way ANOVA with a post hoc Tukey test; $p<0.0001$. All points displayed; red lines indicate mean ±SE.

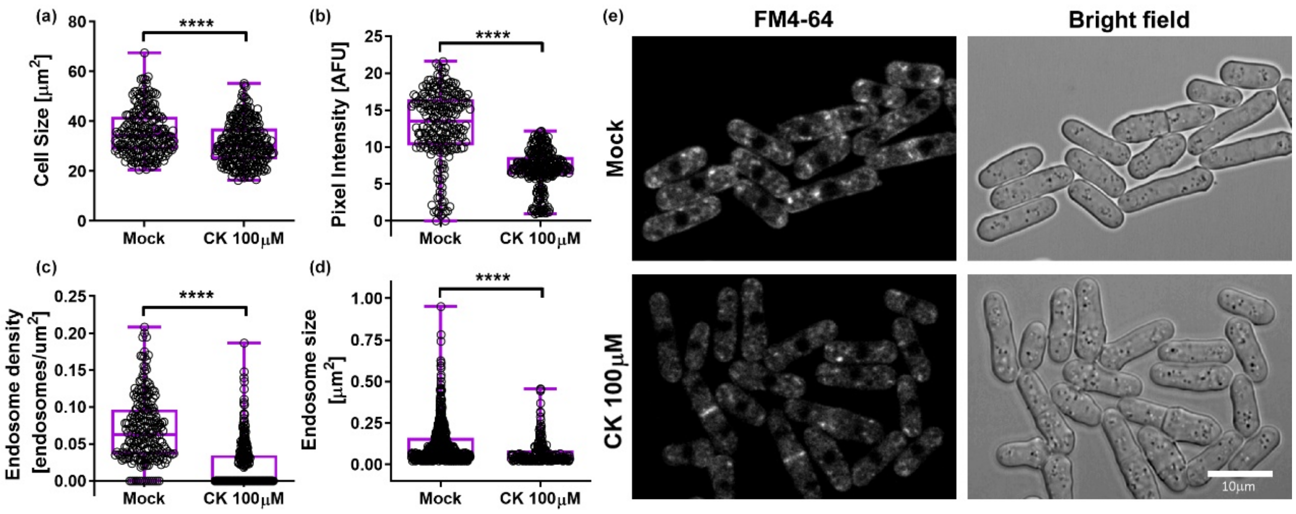

Fig. S6B. Cytokinin inhibits FM-4-64 endocytosis in fission yeast.

S. pombe were grown overnight at 30°C in YE medium, diluted (OD_{600} = 0.2) and incubated for 6 hours in YE medium (Mock) or medium supplemented with 100 µM CK (6-Benzylaminopurine). Cells were incubated with 24 µM FM4- 64 (Invitrogen) at 4°C for 30 min. Subsequently, the FM4-64 containing medium was replaced with fresh medium and cultures was incubated at 28°C for 15 minutes. Confocal microscopy images were acquired using a Zeiss LSM780 confocal microscope system with Objective LD C-Apochromat 63×/1.15 Corr. Acquisition settings were designed using an excitation laser wavelength of 514 nm (4% power). The emission was then collected in the range of 592–768 nm. Bright field was acquired using the T-PMT (transmitted light detector). **(a)** cell size; **(b)** total internalized FM4-64 per cell represented by pixel intensity; **(c)** endosome density; **(d)** endosome size. **(e)** Representative images, Bar, 10 µm. Box-plots with all values displayed; line indicates median. **a-c:** N>220; **d:** N>180. Image analysis was performed using Fiji-ImageJ with raw images collected from 3 independent biological experiments. Endosome count and size measurements were done with the 3D Object counter tool and pixel intensity was measured using the measurement analysis tool. Asterisks represent statistical significance in a Mann-Whitney U test, ****p<0.0001.
