# Supplementary material for: Cytokinin Inhibits Fungal Development and Virulence by Targeting the Cytoskeleton and Cellular Trafficking
Source: mBio. 2021 Oct 19;12(5):e03068-20. doi: 10.1128/mBio.03068-20 (PMC8524340; doi:10.1128/mBio.03068-20)
Supplement: TABLE S1 [file mbio.03068-20-st001.pdf]

Supplementary Table 1
Solvent gradients and MS-MS parameters for CK quantification.

Solvent gradient program for cytokinins:

Time (min)	Phase A %	Phase B %
Initial	95	5
0.5	95	5
14	50	50
15	5	95
18	5	95
19	95	5
22	95	5

LC-MS-MS parameters for quantifications:

Analite and IS	Retention time (min)	Ionization Mode	MRM transition (m/z)	Dwell time (msec)	Cone (V)	Collision (V)
t-Z	2.34	positive	220>202<br>220>136	78	26	14<br>18
^{2}H5-t-Z	2.35	positive	225>137<br>225>207	78	22	18<br>12
iP	4.86	positive	204>136<br>204>69	30	20	14<br>16
^{2}H6-iP	4.82	positive	210>137<br>210>75	30	24	16<br>18
