# Supplementary material for: Cytokinin Inhibits Fungal Development and Virulence by Targeting the Cytoskeleton and Cellular Trafficking
Source: mBio. 2021 Oct 19;12(5):e03068-20. doi: 10.1128/mBio.03068-20 (PMC8524340; doi:10.1128/mBio.03068-20)
Supplement: TABLE S2 [file mbio.03068-20-st002.pdf]

Supplementary Table 2A

Primers used in RT-qPCR.

Gene	Sequence (5'-3')	Primer Efficiency
Tubulin alpha	F- TCTTGCTTCGAGCCCAAC<br>R- ACAGCAGCGTGGACATCA	0.96
pfy1	F- ACCGCCGAGACTGTTCAA<br>R- CCCACCACACATCATCCA	0.96
aft1	F- ATCTGGCTGGTGGCACAT<br>R- CCCACCACACATCATCCA	1.00
sac6	F-CCTCAACCTGCCTCCTGA<br>R-TCGCTCCCTTGTGTAGCC	1.02
smt	F-AGCACCAAACTCCGTTCG<br>R-TAACTGCCAACGCAACGA	1.02
Ub (Ubiquitin)	F-CATCAACTCCAACGGAAGCA<br>R-TCGGTCGGTCTTGTAAACGT	1.00
Iron transport multicopper oxidase	F-GTTTTTGGGACCGGCTTT<br>R-GCCGCTTTTGAGGGAAAT	0.99
Adenosine deaminase	F-TGAGTGCCACGACGAAAA<br>R-ACTCCACCATTGCCTCCA	1.02

Supplementary Table 2B

Oligonucleotides used for generating and validating *Saccharomyces cerevisiae* mutant strains.

Primer	Sequence (5’-3’)
YPT31_Disruption_Fw	GAATAACAATTTGACCTTTATTACAAGGCACTTT GTTTAGGCCAGCAAAGGGATTCTGACGGCGTCT GGGGATTTCAACAcggatccccgggttaattaa
YPT31_Disruption_Rev	AAAATTGTAAAAATATAGCACAGAATTAAAGGG GAGAAGAGTCATTCACATGCAAGTGCGCAACT GCTGCAAAATATCTCgaattcgagctcgtttaaac
YPT31_val_Fw	GTGCGGGTGCTAAATTAGAGA
YPT31_val_Rev	GATGAAGACGAAGAAGACGATG
SSA1_Disruption_Fw	TCTATTTGTAAGATAAGCACATCAAAAGAAAAG TAATCAAGTATTACAAGAAACAAAAATTCAAGT AAATAACAGATAATcggatccccgggttaattaa
SSA1_Disruption_Rev	AAAAACGTTCGGAAAATTCCTCATTATACCCAG ATCATTAAAAGACATTTTCGTTATTATCAATTGCC GCACCAATTGGCgaattcgagctcgtttaaac
SSA1 _val_Fw	CTTCGAGAAGGGATTGAGTTG
SSA1 _val_Rev	GTAGCAGTACTTCAACCATTAG
VPS1_Disruption_Fw	TAAAAAAGAATTAGAGAGGCCTTTTATAGC ACCAAAATAAGGACCGTACGAAAACTGCACATT TTATATT ATCAGATATCcggatccccgggttaattaa
VPS1_Disruption_Rev	CAATATATAAGATTTGCAGTAAATATTAGGGAGA AATACTCAAAACCAAGCTTGAGTCGACCGGTAT AGATGAGGAAAACgaattcgagctcgtttaaac
VPS1_val_Fw	CGTCGCTTTGCCATCAAGAGA
VPS1_val_Rev	AGACTAGCTTCCACGTATAC
SPO14_Disruption_Fw	CGACCGGTCACTGATAATTCACACGACGCATTG AGAGGCACGTACGCAAGAAGAAAAGGTAGGAT AGATAAACAAGGGTGcggatccccgggttaattaa
SPO14_Disruption_Rev	TATGTATCAGCGTCGAATGCTTATAACAGATAAA AGGAAAATACAGGTAATGGTGTGTTCCTGGTCG TTTTTATATTCCCgaattcgagctcgtttaaac
SPO14_val_Fw	GCAGGAC ATTATAGGCA CGA
SPO14_val_Rev	GCAATAATGACACTATGGACC

Supplementary Table 2C

Oligonucleotides used for generating and validating *Botrytis cinerea* mutant strains.

Primer	Sequence (5’-3’)
GA 34F	CGGGTGAATGGGATTCATTG
GA 34R	GCCCGCATTGGATTAATAATTG
GA 44F	GCCACAGACTCCGCCAGATTCTAATG
GA 44R	CAACCATTTCACGCTGCGACCACC
GA 31F	GCAACTAGTGATATTGAAGG
GA 31R	CATCTACTCTATTCCTTTGC
